# Late blight pathogen targets host Rab‐G3 GTPases with an atypical GTPase‐activating protein

**DOI:** 10.1111/jipb.13920

**Published:** 2025-05-07

**Authors:** Song Liu, Liwen Ding, Xiong Liu, Xiaoxi Xing, Jinyang Li, Tiantian Yan, Yuli Huang, Yuan Liu, Yisa Wang, Xia Zhang, Zeming Liu, Xiyu Cao, Yuling Meng, Weixing Shan

**Affiliations:** ^1^ State Key Laboratory of Crop Stress Resistance and High‐Efficiency Production and College of Agronomy Northwest A&F University Yangling 712100 China

**Keywords:** GTPase‐activating protein (GAP), NbRab‐G3c, *Phytophthora infestans*, Pi17063, Ras‐related brain (Rab) GTPase, RXLR effector

## Abstract

Late blight pathogen *Phytophthora infestans* secretes numerous effectors to suppress plant immunity. However, little is known about their underlying biochemical mechanisms. Here we report that, in the host *Nicotiana benthamiana*, *P. infestans* core RXLR effector Pi17063 suppresses plant immunity by targeting the host plasma membrane and NbRab‐G3 proteins, small GTPases of the Ras‐related brain (Rab) family. Pi17063 functions as their specific GTPase‐activating protein (GAP), driving them to the cytoplasm‐localized guanosine diphosphate (GDP)‐bound inactive state. Mutant analysis of the conserved Pi17063 arginine residues showed the essential role of its GAP activity for virulence contribution. All four NbRab‐G3 subfamily members are positive immune regulators, and NbRab‐G3c mutants lost the ability to switch between active and inactive states and showed compromised immune function. Consistent with this, both silencing and overexpression of an endogenous GAP, *NbGYP*, inhibited NbRab‐G3c‐mediated plant immunity. Our results revealed positive immune roles of host NbRab‐G3 GTPases, the importance of their state balance, and the biochemical mechanism by which their functions are suppressed by a *P. infestans* effector, providing insights into understanding eukaryotic effector‐mediated plant susceptibility.

## Graphical abstract

The *Phytophthora infestans* RXLR effector Pi17063 functions as an atypical GTPase‐activating protein of host positive immune regulators, specifically the small GTPase NbRab‐G3. Pi17063 promotes the relocalization of NbRab‐G3 proteins to the cytoplasm, where they are inactive, disrupting the balance of the NbRab‐G3 switch and thus leading to enhanced plant susceptibility.

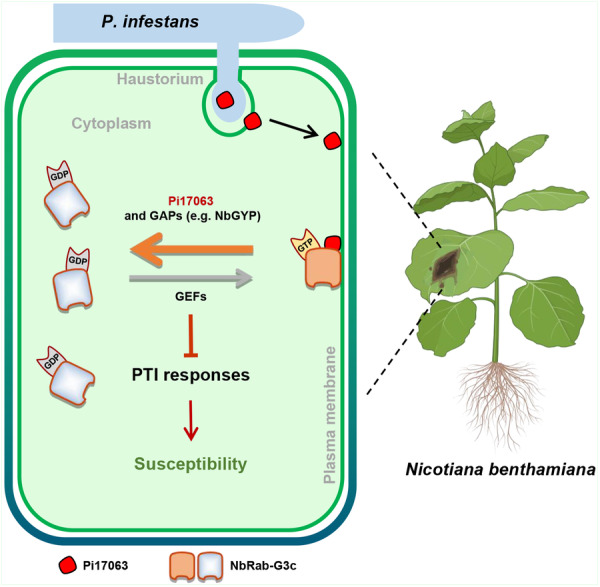

## INTRODUCTION

Oomycetes are a class of eukaryotes that are evolutionarily close to algae and occupy a unique branching position on the Tree of Life (Baldauf, 2003). Many oomycetes are plant pathogens and cause severe diseases, which pose serious threats to agricultural production and natural ecosystems (Kamoun et al., 2015). Potato late blight, caused by *Phytophthora infestans*, is the most devastating disease of potato and tomato (Tian et al., 2015).

Plants are endowed with complex, sensitive immune systems to protect them from microorganisms and insects. Accordingly, pathogen recognition, signal integration, and defense functions are strictly regulated (Wang et al., 2019a). The immune responses of plants can be generally divided into pattern‐triggered immunity (PTI) and effector‐triggered immunity (ETI) based on the molecular method of pathogen recognition (Jones and Dangl, 2006). Pattern recognition receptors (PRRs) are present on host plasma membranes and can monitor and recognize pathogen‐associated molecular patterns (PAMP), providing basal immune responses such as reactive oxygen species (ROS) burst, callose deposition and expression of defense‐related genes. Another group of plant immune receptors are nucleotide‐binding leucine‐rich repeat receptors (NB‐LRRs), which recognize various effectors (Jones and Dangl, 2006). PTI and ETI have a great deal of overlap in signaling networks and downstream responses (Ngou et al., 2021; Yuan et al., 2021).

During host interactions, *P. infestans* secretes numerous RXLR effectors with variable sequences and diverse targets to interfere with host plant immune responses. RXLR effectors consist of a signal peptide at the N‐terminus that is ~20 amino acids in length, followed by a conserved RXLR (arginine‐X‐leucine‐arginine)‐dEER motif (Rehmany et al., 2005). RXLR effectors target a variety of host proteins to disrupt defense responses (He et al., 2020). For example, they are documented to interfere with signal transduction (King et al., 2014), regulate host transcription (Qiao et al., 2015), modulate the activity of related enzymes (Lin et al., 2021), disturb host metabolism (Yang et al., 2020) and alter host protein stability (Du et al., 2021).

Molecular modules involved in cell signaling, such as small GTPases, are highly conserved and play irreplaceable roles in plants (Nielsen, 2020). Small GTPases are a class of 21–30 kDa proteins that bind to single guanine nucleotides (Rutherford and Moore, 2002). In plants, small GTPases can be divided into four families: Ras‐related nuclear (Ran), Ras‐related brain (Rab), ADP‐ribosylation factor (Arf) and Rho of plant (ROP) GTPases. In *Arabidopsis thaliana*, there are 57 Rab GTPases that can be divided into eight subfamilies (RabA–RabH) (Vernoud et al., 2003). *A. thaliana* encodes eight putative Rab‐G GTPases, which are reported to localize to the vacuolar and plasma membranes (Saito and Uedat, 2009). They are involved in a range of processes, including vacuole biogenesis (Rojo et al., 2001), autophagy (Kwon et al., 2010) and immunity‐associated hypersensitive cell death (Kwon et al., 2013).

Small GTPases exist in two states, the active guanosine triphosphate (GTP)‐bound state, in which the small GTPases localize on endomembrane and bind to downstream signaling effectors to regulate intracellular processes, and the cytoplasm‐localized inactive guanosine diphosphate (GDP)‐bound state. The subcellular localization of small GTPases is dynamic, as they switch between active and inactive states. The states of small GTPases are strictly regulated (Nielsen et al., 2008). GTPase‐activating proteins (GAPs) bind to small GTPases in an active state, activating them and promoting GTP hydrolysis. This process converts small GTPases from the active to the inactive state. In most eukaryotes, Rab GAPs are composed of two subdomains that together surround the Rab GTPase G‐domains (Pylypenko et al., 2018). GAP function is dependent on a dual *trans*‐finger mechanism. In this process, the conserved arginine residues are irreplaceable (Pan et al., 2006). Intracellular guanine nucleotide exchange factors (GEFs) can alter the conformation of small GTPases in the inactive state, promoting their dissociation from GDP and binding of GTP, which switches the small GTPases to the active state. In plants, GAPs (Yuen et al., 2024) and GEFs (Nielsen et al., 2017) are essential for the function of small GTPases and are involved in the regulation of plant responses to biotic and abiotic stresses.

Rab GTPases have been shown to play critical roles in plant immunity through processes such as trafficking and recycling of receptors (Choi et al., 2013) and defense components (Bozkurt et al., 2015; Tomczynska et al., 2018), cytoskeleton rearrangement (Hoefle et al., 2011), and regulation of defense‐related genes (Speth et al., 2009). Similarly, pathogens have evolved diverse strategies to interfere with the function of small GTPases. For example, bacterial effectors from *Shigella flexneri* (Dong et al., 2012), *Salmonella* (Spanò et al., 2016) and *Legionella pneumophila* (Mukherjee et al., 2011; Tan et al., 2011) have been reported to target and directly alter the state of host Rab GTPases, subsequently affecting host cell immunity. Effectors secreted by fungal and oomycete pathogens also target the plant vesicular trafficking system to interfere with immune processes (Petre et al., 2021). However, the small secreted fungal and oomycete effector proteins, unlike bacterial effectors, lack typical enzymatically active domains (He et al., 2020). Therefore, little is known about the role and underlying biochemical mechanisms of fungal and oomycete effectors in suppressing small GTPase‐mediated immunity, although there have been a few reports on the host small GTPases targeted by oomycete effectors (Tomczynska et al., 2018; Li et al., 2022).

Here, we report that the host NbRab‐G3 GTPases are plasma membrane‐localized positive immune regulators in *N. benthamiana*. The *P. infestans* core RXLR effector Pi17063 functions as an atypical GAP of NbRab‐G3 proteins, leading to their cytoplasm‐localized inactive state (GDP‐bound). The GAP activity of Pi17063 is indispensable for its virulence function. Analysis of the endogenous GAP NbGYP and of the NbRab‐G3c mutants locked in active or inactive state show that the ability of NbRab‐G3c to switch between two states is essential for its immune function. Taken together, our results reveal the positive immune roles of host NbRab‐G3 GTPases, the importance of their state balance, and the biochemical mechanisms by which their functions are suppressed by the late blight effector Pi17063, leading to enhanced plant susceptibility.

## RESULTS

### Host plasma membrane localization of *P. infestans* effector Pi17063 is required for its virulence function

**Figure 1:**
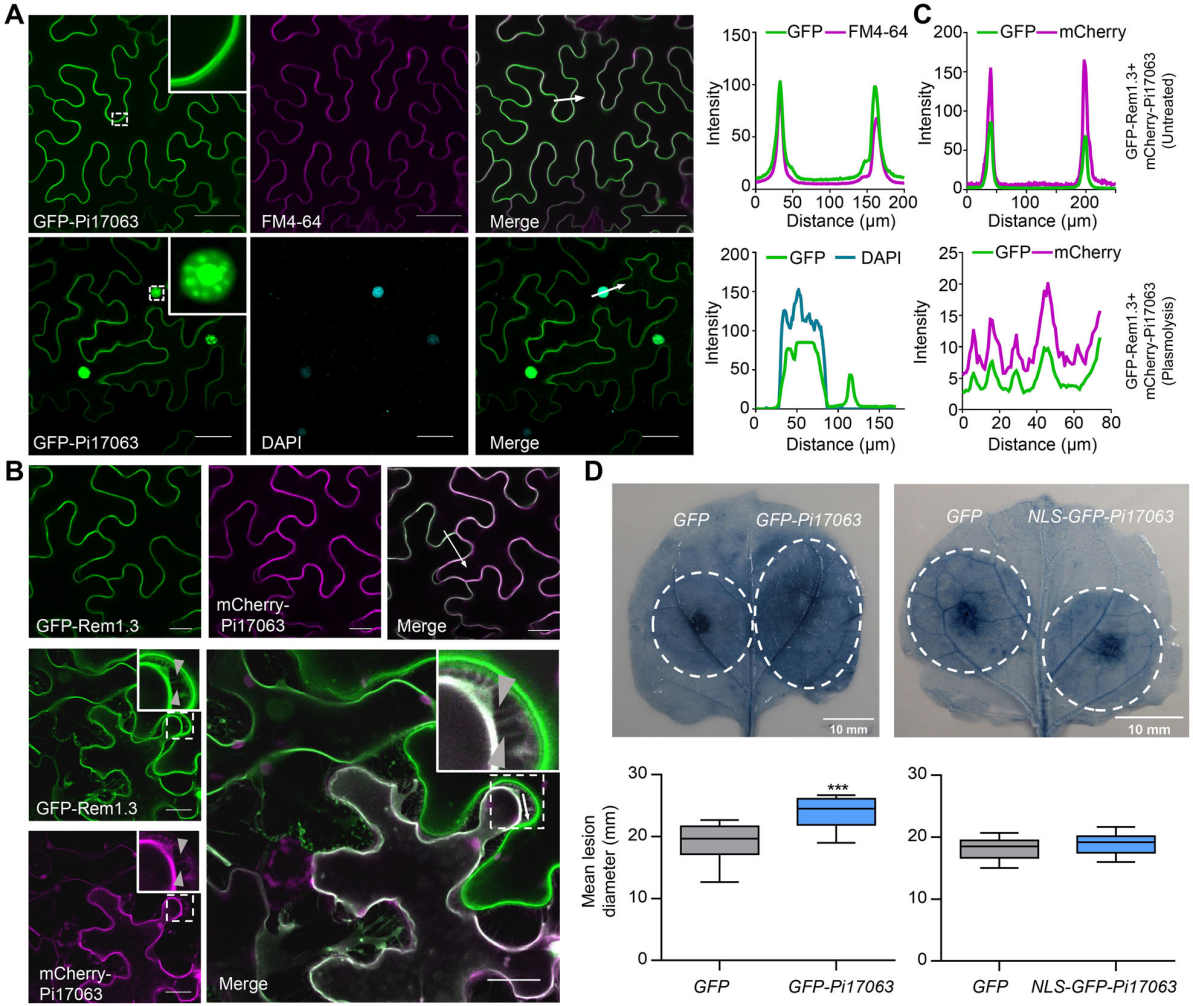
Host plasma membrane (PM) localization of Pi17063 is indispensable for its virulence function **(A**, **B)** Host plasma membrane and nuclear speckle localization of Pi17063. Scale bars = 40 μm **(A)** or 20 μm **(B)**. The white dashed squares indicate local zoom‐in insets. Examples of hechtian strands are highlighted with gray‐filled triangles. **(C)** Fluorescence intensity was quantified along the transects (white line) using ImageJ software. **(D)** Infection assays on *GFP‐Pi17063* or *NLS‐GFP‐Pi17063* transiently expressed leaves. *N. benthamiana* leaves were inoculated with *P. infestans* zoospores at 1 dpi. *GFP* was uses as a negative control. Leaves were stained with trypan blue to highlight lesions. Lesion diameters are quantified from 14 independent leaves. Dotted white circles indicate lesion areas. The upper quartile, median, and lower quartile are shown in each box plot, while the bars outside the box indicate the maximum and minimum values. ***P < 0.001 (Student's *t*‐test).

Pi17063 was previously defined as a candidate core RXLR effector of *P. infestans* because it is upregulated at early infection stages and its protein sequence is highly conserved between multiple *P. infestans* strains (Yin et al., 2017). To further investigate its function, we examined the host subcellular localization of Pi17063. *Green fluorescent protein (GFP)‐Pi17063* was expressed in *N. benthamiana* leaves, which were stained with fluorescent dyes FM4‐64 (for the plasma membrane [PM]) or 4',6‐Diamidino‐2‐Phenylindole (DAPI), for the nuclei) before the confocal microscopic observation was conducted. GFP‐Pi17063 fluorescence coincided with both FM4‐64 and DAPI (Figure 1A), indicating that Pi17063 was both a PM‐ and a nucleus‐localized effector. To further confirm its PM localization, *Pi17063* was co‐overexpressed with *GFP‐Rem1.3* or *mCherry‐γ‐TIP* in *N. benthamiana* leaves. The former is a PM‐localized marker (Bozkurt et al., 2015) and the latter is a vacuolar membrane‐localized aquaporin (Nelson et al., 2007). Their subcellular localizations were clearly distinguishable (Figure S1A). Confocal observations showed that Pi17063 was clearly co‐localized with Rem1.3 on the PM (Figure 1B, C) but not with *γ*‐TIP on the vacuolar membrane (Figure S1B). A plasmolysis analysis showed clear hechtian strands of Pi17063 (Figures 1B, S1C), further confirming its PM localization.

Pi17063 can render the host plant *N. benthamiana* more susceptible to *P. infestans* colonization (Yin et al., 2017; Wang et al., 2019b). To determine whether the PM‐ or nucleus‐localization of Pi17063 is required for its function, we fused a nuclear localization signal (NLS) to the N‐terminus of GFP‐Pi17063 (NLS‐GFP‐Pi17063) and transiently expressed this construct in *N. benthamiana* leaves by agroinfiltration. Confocal microscopy showed that NLS‐Pi17063 was re‐localized to the nucleus (Figure S2A), and protein expression and integrity were confirmed via western blot (Figure S2B). A *P. infestans* zoospores infection assay showed that *GFP‐Pi17063* overexpression led to a significant increase in both lesion size and *P. infestans* colonization, whereas *NLS‐GFP‐Pi17063* overexpression could not (Figures 1D, S2C, D). Consistent with this result, a luminol‐based chemiluminescence assay also showed that nuclear‐localized Pi17063 abolished its function in inhibiting plant PTI responses (Figure S2E). Taken together, these results indicated that Pi17063 requires host PM localization to exert its virulence function, and that the N‐terminal GFP fusion does not affect Pi17063 function.

### Pi17063 specifically targets NbRab‐G subfamily small GTPases

**Figure 2:**
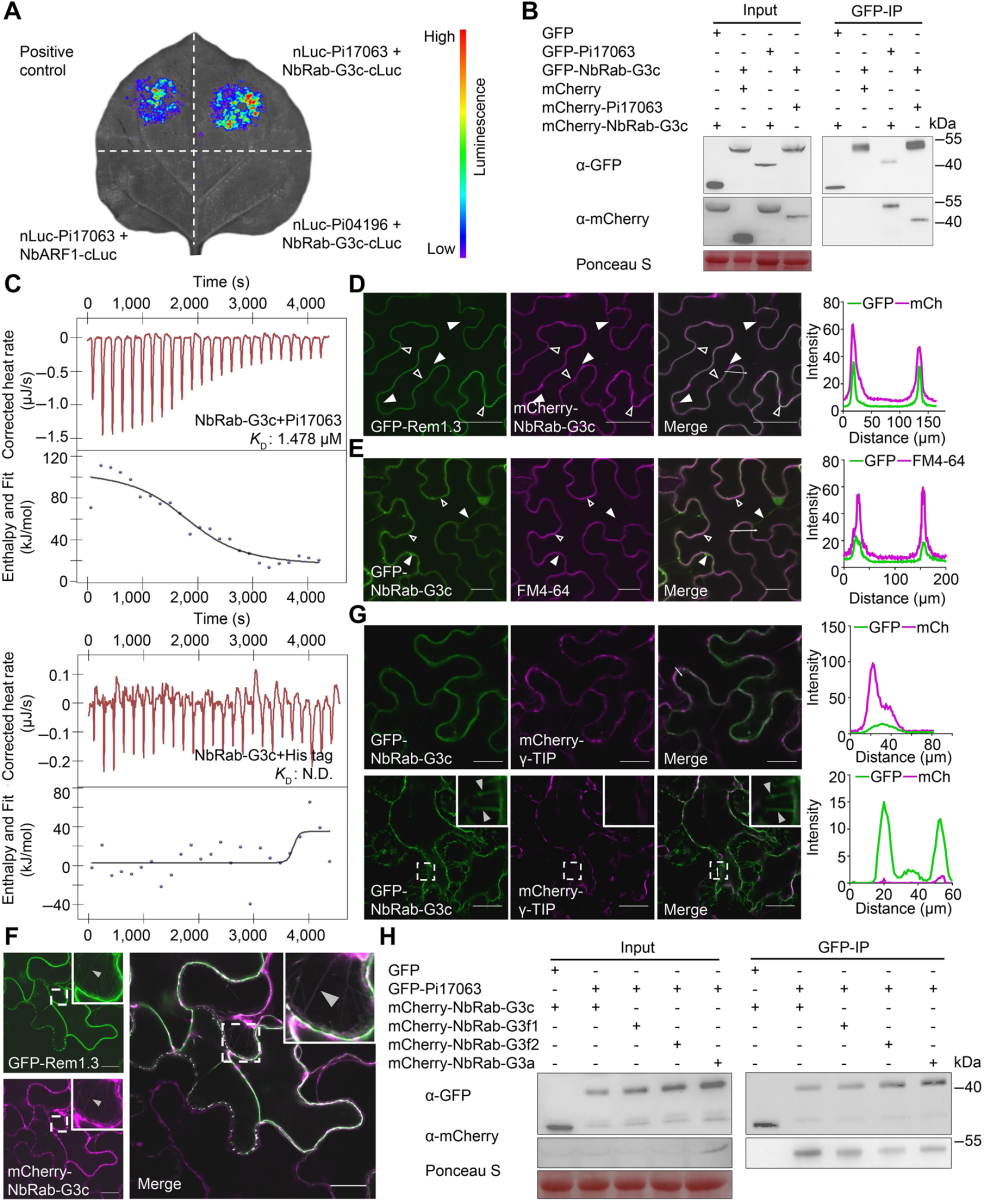
Pi17063 interacts with the plasma membrane (PM)‐localized NbRab‐G3c **(A)** Luciferase complementation assays were used to indicate the interaction between Pi17063 and NbRab‐G3c. nLuc‐Pi17063 + NbARF‐cLuc, nLuc‐Pi04196 + NbRab‐G3c‐cLuc were used as the negative controls. cLuc‐RbohD + BIK1‐nLuc was set as the positive control. Leaf images were taken at 3 dpi. **(B**, **H)** Co‐IP assays using GFP‐trap beads. Total protein was extracted from *N. benthamiana* leaves at 3 dpi. Co‐precipitation was detected via western blotting. Ponceau S staining indicates protein samples loaded. **(C)** Pi17063 interacts with NbRab‐G3c *in vitro*. Recombinant NbRab‐G3c protein was titrated with identical concentrations of Pi17063 and His tag proteins in isothermal titration calorimetry assays. *K*_D_, dissociation constant; N. D., Not Detectable. **(D)** Subcellular localization of mCherry‐NbRab‐G3c. Confocal observations were performed at 3 dpi, with GFP‐Rem1.3 as the PM marker. **(E)** PM localization of NbRab‐G3c as indicated by FM4‐64‐staining before confocal observation. **(F)** Co‐localization of GFP‐Rem1.3 and mCherry‐NbRab‐G3c on hechtian strands. **(G)** Localization of NbRab‐G3c relative to vacuolar membranes. Confocal images information: Scale bars = 40 μm **(D)** or 20 μm **(E**–**G)**. Fluorescence intensity was quantified along the transects (white line) using ImageJ software. The white dashed squares indicate local zoom‐in insets. Examples of PM, cytoplasmic and hechtian strand localizations are highlighted with open, white‐filled and gray‐filled triangles, respectively.

To further understand Pi17063‐mediated plant susceptibility, we screened for interacting host targets using a liquid chromatography‐tandem mass spectrometry (LC–MS/MS) assay. In total, 37 candidate target proteins were identified to be present in the LC–MS/MS results using GFP‐Pi17063 but absent in the negative control screen with GFP (Table S1). Luciferase complementation imaging (LCI) assay (Zhou et al., 2018) was used to test the interactions between Pi17063 and its candidate targets. This led to the identification of the small GTPase NbRab‐G3c (Niben101Scf01374g03034.1) (Figure 2A), which is a Ras‐related brain (Rab) GTPase family member. To further confirm their interaction, we generated the fusion constructs *GFP‐NbRab‐G3c* and *mCherry‐Pi17063*. The infection assay showed that fusion had no impact on the ability of Pi17063 to promote infection (Figure S3). The co‐immunoprecipitation (co‐IP) assay further confirmed the interaction between Pi17063 and NbRab‐G3c (Figure 2B). Moreover, the isothermal titration calorimetry (ITC) assay with purified recombinant proteins also showed that Pi17063 bound with NbRab‐G3c *in vitro* (Figure 2C).

The subcellular localization of small GTPases is crucial for understanding its function (Nielsen, 2020). To determine the subcellular localization of NbRab‐G3c, *mCherry‐NbRab‐G3c* was co‐overexpressed in *N. benthamiana* leaves with *GFP‐Rem1.3*. Confocal observation showed that mCherry‐NbRab‐G3c mostly co‐localized with GFP‐Rem1.3 on the PM, together with some cytoplasmic localization (Figure 2D). The partly PM localization of NbRab‐G3c was also confirmed by FM4‐64 staining (Figure 2E). A plasmolysis analysis showed co‐localization of NbRab‐G3c with Rem1.3 as indicated by clear hechtian strands (Figures 2F, S4A) but not with γ‐TIP (Figure 2G), excluding its possible vacuolar membrane localization. NbRab‐G3c thus exhibited dual localization (PM and cytoplasm), which is consistent with the characteristics of small GTPases.

**Figure 3:**
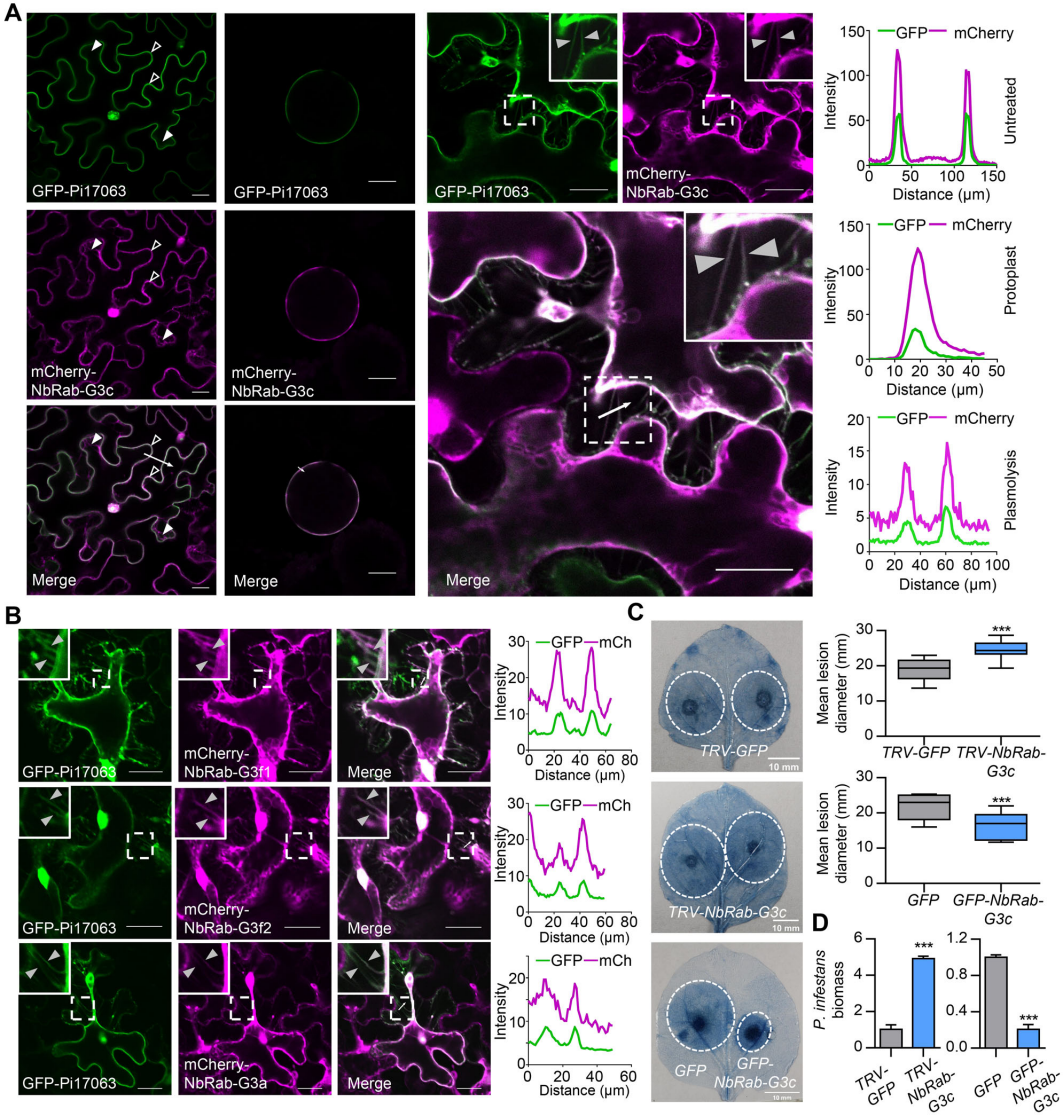
NbRab‐G3 proteins co‐localize with Pi17063 on plasma membrane (PM) and positively regulate plant immunity **(A)** Co‐localization of GFP‐Pi17063 and mCherry‐NbRab‐G3c. Images were taken from untreated (left panels), leaf protoplasts (middle panels) or 2 M NaCl‐induced plasmolyzed (right panels). **(B)** Co‐localization of GFP‐Pi17063 with mCherry‐NbRab‐G3 proteins. **(C)** *N. benthamiana* leaves with *GFP‐NbRab‐G3c* and *GFP* overexpressed (10 biologically independent samples), with *GFP* or all *NbRab‐G3* genes silenced (9 biologically independent samples), were inoculated with *P. infestans* zoospores. Leaves were stained with trypan blue. Dotted white circles indicate lesion areas. The upper quartile, median, and lower quartile are shown in each box plot, while the bars outside the box indicate the maximum and minimum values. ***P < 0.001 (Student's *t*‐test). **(D)** *P. infestans* biomass in *N. benthamiana* leaves. Data are presented as the mean ± standard error (*n* = 3). ***P < 0.001 (Student's *t*‐test). Confocal images information: Scale bars = 20 μm **(A**, **B)**. Fluorescence intensity was quantified along the transects (white line) with ImageJ. The white dashed squares indicate local zoom‐in insets. Examples of PM, cytoplasmic and hechtian strand localizations are highlighted with open, white‐filled and gray‐filled triangles, respectively.

Therefore, we further determined the subcellular co‐localizations of mCherry‐NbRab‐G3c and GFP‐Pi17063 via agroinfiltration‐mediated co‐expression in *N. benthamiana* leaves. Their co‐localization was confirmed in *N. benthamiana* epidermal cells and in protoplasts (Figure 3A). In addition, their PM co‐localization was further indicated by the presence of hechtian strands (Figure 3A). Moreover, we swapped the fluorescent tags of these two proteins, and the co‐localization of GFP‐NbRab‐G3c and mCherry‐Pi17063 was also confirmed by confocal microscope observation (Figure S4B). Protein expression and integrity were confirmed via western blot (Figure S4C). To further confirm that Pi17063 interacts with NbRab‐G3c on PM, a bimolecular fluorescence complementation (BiFC) assay was performed. Another *P. infestans* effector Pi23042 (Yin et al., 2017), with typical PM localization (Figure S5A, B), was used as a negative control. The results showed that co‐expression of NbRab‐G3c‐YFP^N^ and Pi17063‐YFP^C^ formed clear fluorescence on PM with or without plasmolysis (Figure S5C, D), indicating their interaction on PM. Taken together, our results demonstrated that NbRab‐G3c has dynamic subcellular localizations between PM and cytoplasm, and co‐localizes and interacts with Pi17063 on the PM.

The plant Rab GTPase family is the largest small GTPase family in plants (Nielsen, 2020). To explore the specificity of the interaction between Pi17063 and NbRab GTPases, we selected, cloned and examined six *NbRab* GTPase genes from the *NbRab‐A*, *NbRab‐B*, *NbRab‐D*, *NbRab‐E* and *NbRab‐F* subfamilies: *Niben101Scf00270g12012.1* (*NbRab‐A5e*), *Niben101Scf01517g08034.1* (*NbRab‐B1b*), *Niben101Scf00684g00002.1* (*NbRab‐E1c1*), *Niben101Scf05032g00006.1* (*NbRab‐D1*), *Niben101Scf09596g00001.1* (*NbRab‐E1c2*), and *Niben101Scf00271g01020.1* (*NbRab‐F2a*) (Figure S6A). All six members showed part co‐localization with Pi17063 on PM, and substantial cytoplasmic localization (Figure S6B, C). Their physical interactions were next tested by co‐IP assays, with results showing no stable interactions between these six NbRab GTPases and Pi17063 (Figure S6D).

The protein sequences of the *N. benthamiana* Rab‐G3 subfamily GTPases are highly conserved (Figure S7A). To explore whether Pi17063 interacted with NbRab‐G3c specifically, we cloned three additional *NbRab‐G3* subfamily members identified through the BLAST tool of the Solanaceae Genomics Network (https://solgenomics.net/tools/blast), *Niben101Scf04778g00002.1*, *Niben101Scf06726g00033.1*, and *Niben101Scf08526g01012.1*, designated as *NbRab‐G3f1*, *NbRab‐G3f2*, and *NbRab‐G3a*, respectively, based on evolutionary analysis with the Rab‐G subfamily protein members in *A. thaliana* (Figure S6A). The results of LCI (Figure S7B) and co‐IP (Figure 2H) assays showed that Pi17063 interacted with all three of these additional NbRab‐G3 members. Confocal observation showed that all tested NbRab‐G3 subfamily members had a dynamic subcellular localization between PM and cytoplasm and co‐localized with Pi17063 on the PM (Figure S8). Co‐localization of NbRab‐G3 proteins with Pi17063 was evident, as revealed by the clear fluorescent hechtian strands after plasmolysis treatment (Figure 3B).

### NbRab‐G3 subfamily members positively regulate plant resistance and PTI responses

To investigate the role of *NbRab‐G3* subfamily genes in plant immunity, we examined their expression patterns in response to *P. infestans* infection. Because of the highly conserved sequences of *NbRab‐G3* subfamily members (Figure S7A), we selected a conserved region and tested the transcript level of these four members. The aggregate transcript levels of *NbRab‐G3* genes were upregulated during early infection (Figure S9A), suggesting their potential role in mediating interactions between *N. benthamiana* and *P. infestans*. Transcriptome data of *N. benthamiana* leaves infected with *P. infestans* at different time points also showed that all four genes were upregulated at the early stages of plant infection (Figure S9B). To further confirm the immune functions of *NbRab‐G3* subfamily genes, we performed an infection assay. The results showed smaller lesions and less *P. infestans* colonization in leaves overexpressing *NbRab‐G3* genes compared with the *GFP* control (Figures 3C, D, S9C). Protein expression and integrity were confirmed via western blot (Figures S9D, S10C). Collectively, these results indicated that these *NbRab‐G3* subfamily genes could positively regulate plant immunity.

The immune function of the *NbRab‐G3* genes was further analyzed using a tobacco rattle virus (TRV)‐based virus‐induced gene silencing (VIGS) assay. As mentioned above, the sequences of *NbRab‐G3* subfamily members are highly conserved, so we designed a single 300‐bp fragment to silence all four members (*NbRab‐G3c, NbRab‐G3f1, NbRab‐G3f2* and *NbRab‐G3a*) in subsequent experiments (Figure S7A). A pair of primers was designed from a highly conserved fragment (Figure S7A) to detect the collective silencing efficiency of *NbRab‐G3* subfamily genes (primers suitable for the assay of individual genes could not be found due to sequence conservation). Compared with the *GFP*‐silenced control plants, a more than 95% decrease in aggregate *NbRab‐G3* transcript levels was notable in the *TRV‐NbRab‐G3* plants, while transcript levels of *NbRab‐A‐NbRab‐F* and *NbRab‐H* were not affected (Figure S10A). The growth of *TRV‐NbRab‐G3* plants was not significantly affected (Figure S10B). After inoculation with *P. infestans*, lesions were larger and colonization was higher in *TRV‐NbRab‐G3* leaves compared with the control (Figure 3C, D). The ROS levels triggered by flg22 were also significantly suppressed in *TRV‐NbRab‐G3* leaves (Figure S10D). To further confirm the role of *NbRab‐G3* genes in plant PTI responses, recombinant protein of *P. infestans* PAMP elicitin INF1 (Kamoun et al., 1998) was prepared (Figure S11A) and directly infiltrated into *N. benthamiana* leaves. Significant hypersensitive response (HR) was observed (Figure S11B), indicating that the recombinant INF1 protein was biologically functional. We then tested the responses of *NbRab‐G*‐silenced plants to INF1 treatment. RT‐qPCR assays showed downregulation of PTI‐related marker genes *WRKY7* and *WRKY8* (Yan et al., 2016) in *TRV‐NbRab‐G3* leaves (Figure S10E). However, the silencing of *NbRab‐G3* genes had no impact on the expression level of salicylic acid (SA) pathway marker gene *PR2* (Yan et al., 2016; Yang et al., 2020) and jasmonic acid (JA) pathway marker gene *PR3* (Yang et al., 2020) (Figure S10E). The silencing results thus indicated that one or more of these four *NbRab‐G3* subfamily genes played positive roles in plant immunity.

### Pi17063 functions as a GAP to promote GTPase activity of NbRab‐G3c

Small GTPases act as molecular switches, changing between the active (GTP‐bound; membrane‐localized) and inactive (GDP‐bound; cytoplasm‐localized) states. As demonstrated above, Pi17063 exhibited clear PM localization, similar to NbRab‐G3c in its active state. To investigate whether Pi17063 functions as a GAP to affect NbRab‐G3c GTPase activity, we purified recombinant Pi17063 and NbRab‐G3c proteins (Figure S11C). To confirm the biological function of recombinant Pi17063 protein, its elicitor activity was analyzed via agroinfiltration, with results showing that it triggers a potato genotype‐specific cell death on the potato cultivar Longshu10 (Figure S11D). The recombinant Pi17063 protein was then infiltrated directly into potato leaves, where it induced an obvious cell death response in Longshu10 (Figure S11D), indicating that our purified recombinant effector protein was biologically functional.

**Figure 4:**
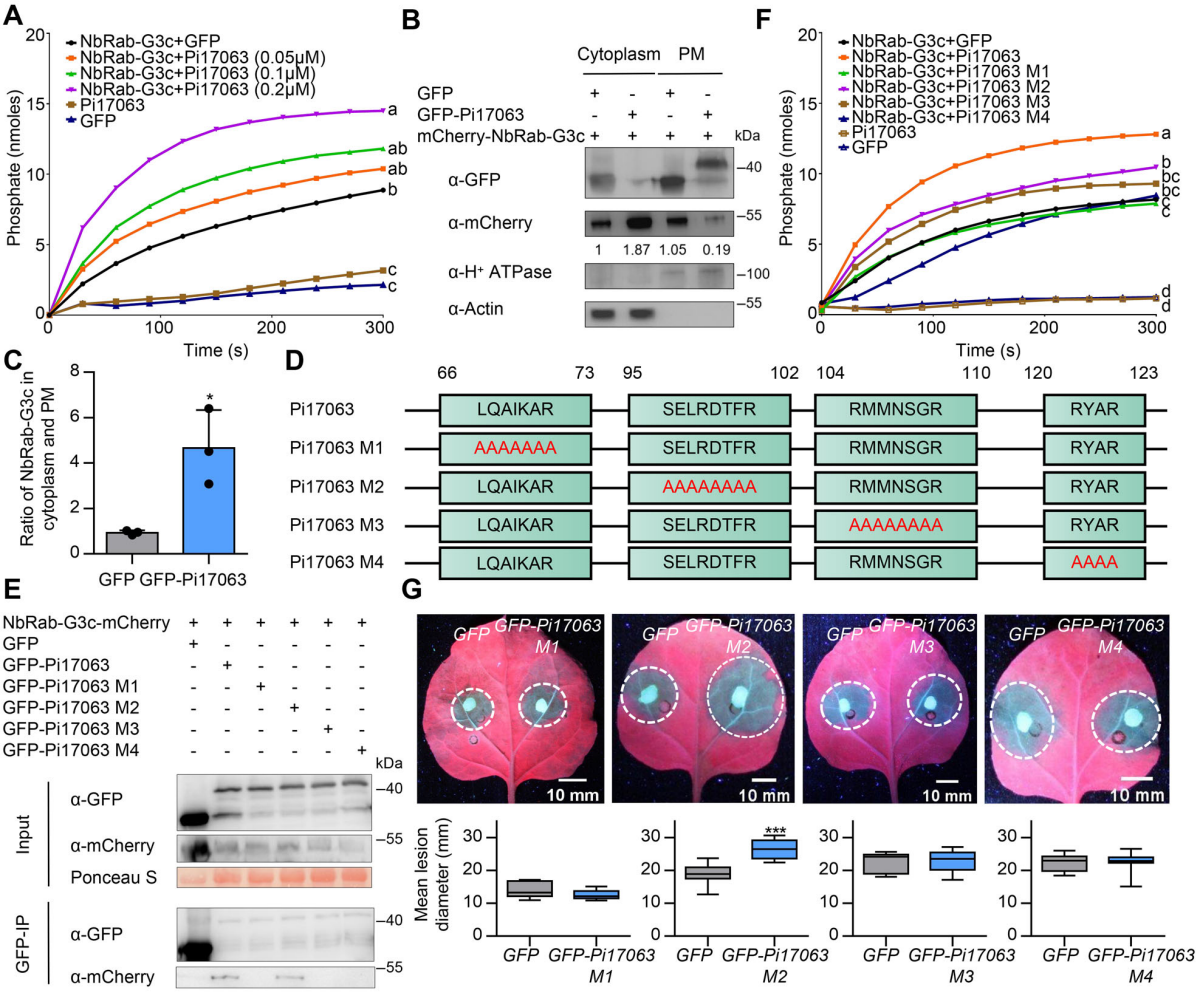
Pi17063 is a GTPase‐activating protein (GAP) **(A)** *In vitro* activation of NbRab‐G3c GTPase activity by Pi17063. Recombinant NbRab‐G3c and Pi17063 proteins were co‐incubated before the GTPase activity was quantified using the ELIPA Biochem Kit (Cytoskeleton, USA), with GFP protein as a negative control. The data at 300 s of reaction were subjected to Tukey's multiple comparisons test; different letters indicate significant differences (*P* < 0.05). **(B**, **C)** Overexpression of Pi17063 reduces plasma membrane (PM)‐localized NbRab‐G3c. Cytoplasm and PM proteins were extracted using the Minute™ Plasma Membrane Protein Isolation Kit from *N. benthamiana* leaves. Protein expression was detected via Western blot, with H^+^ ATPase and Actin as PM and cytoplasm marker proteins, respectively. Gray values were calculated using ImageJ software. *P < 0.05 (Student's *t*‐test). **(D)** Schematic representation of Pi17063 mutant constructs. Red residues indicate mutation sites. **(E)** Co‐IP assays with GFP‐trap beads. Total protein was extracted from *N. benthamiana* leaves at 3 dpi. Co‐precipitation was detected via western blotting. Ponceau S staining indicates amount of protein samples loaded. **(F)** *In vitro* GAP activities of Pi17063 mutant proteins. The data at 300 s of reaction were subjected to Tukey's multiple comparisons test; different letters indicate significant differences (*P* < 0.05). **(G)** Immune function of Pi17063 mutants. *N. benthamiana* leaves expressing each of the derivative *Pi17063* mutants were inoculated with *P. infestans* zoospores. Lesion images were taken under UV light. Data are obtained from 10 independent leaves. Dotted white circles indicate lesion areas. The upper quartile, median, and lower quartile are shown in each box plot, while the bars outside the box indicate the maximum and minimum values. ***P < 0.001 (Student's *t*‐test).

The purified recombinant proteins were co‐incubated and the GTPase activity of NbRab‐G3c was quantitated using a GTPase enzyme‐linked inorganic phosphate assay (ELIPA) Biochem Kit. Co‐incubation of NbRab‐G3c and Pi17063 significantly promoted the Pi release rate compared with that of NbRab‐G3c alone (Figure 4A). As the concentration of Pi17063 increased, there was a marked increase in NbRab‐G3c GTPase activity (Figure 4A), indicating that the hydrolysis activity of NbRab‐G3c was enhanced by Pi17063. These results suggested that Pi17063 enhanced NbRab‐G3c GTPase activity and promoted its switch from the active to the inactive state, meaning that Pi17063 had GAP functionality.

Different states of small GTPases determine their different subcellular localization (Nielsen, 2020). To further confirm the GAP activity of Pi17063, we tested whether it affected NbRab‐G3c subcellular localization. *GFP‐Pi17063* or *GFP* was co‐overexpressed with *mCherry‐NbRab‐G3c* in *N. benthamiana* leaves by agroinfiltration. Cytoplasm and PM proteins were isolated, respectively, and analyzed using western blot assays. The results showed that the expression of Pi17063 significantly increased in the cytoplasmic localization, which is the critical characteristic of small GTPases in an inactive state, of NbRab‐G3c (Figure 4B, C), further confirming the GAP activity of Pi17063.

To determine the relationship between Pi17063 GAP activity and its virulence function, mutant analysis was performed. Pi17063 did not display a recognizable GAP domain, however, the arginine residue has been identified as the key residue of Arg‐finger domains (Pylypenko et al., 2018). So, we searched for regions adjacent to arginine and mutated each region to Ala residues to generate four mutants, Pi17063M1, Pi17063M2, Pi17063M3, and Pi17063M4 (Figure 4D). Of these four regions, analysis of sequences conserved in Pi17063 and its closest homologs in other *Phytophthora* species showed that the arginine residues of the Pi17063M3 and M4 regions were highly conserved (Figure S12A). This conservation further suggested that those arginine residues might play a key role in the function of Pi17063 and its homologs.

Confocal observation showed that the Pi17063M2, Pi17063M3, and Pi17063M4 mutant proteins co‐localized on the PM with NbRab‐G3c (Figure S12B). While Pi17063M1 completely lost PM localization (Figure S12B). Protein integrity was confirmed via western blot (Figure S12C). To determine whether NbRab‐G3c interacts with these four Pi17063 mutant proteins, co‐IP assays showed that only Pi17063M2 retained its interaction with NbRab‐G3c; the other three mutants almost completely lost their ability to target NbRab‐G3c (Figure 4E). Furthermore, we prepared recombinant Pi17063 mutant proteins (Figure S11C) to measure their GAP activity. Compared with wild‐type Pi17063, three of the Pi17063 mutant proteins were attenuated in promoting NbRab‐G3c GTPase activity (Figure 4F). Only Pi17063M2 still promoted NbRab‐G3c GTPase activity at a relatively high level (Figure 4F).

We further tested the virulence functions of these four Pi17063 mutants. *Pi17063M2* overexpression significantly promoted infection and colonization by *P. infestans* to a level comparable to that of wild‐type *Pi17063*, whereas the other three mutants lost their virulence function (Figures 4G, S12D, E). These results indicated that mutations in the conserved arginine residue of Pi17063 decreased GAP activity and abolished the disease‐promoting function.

### NbRab‐G3c‐mediated defense responses depend on its full switching ability

**Figure 5:**
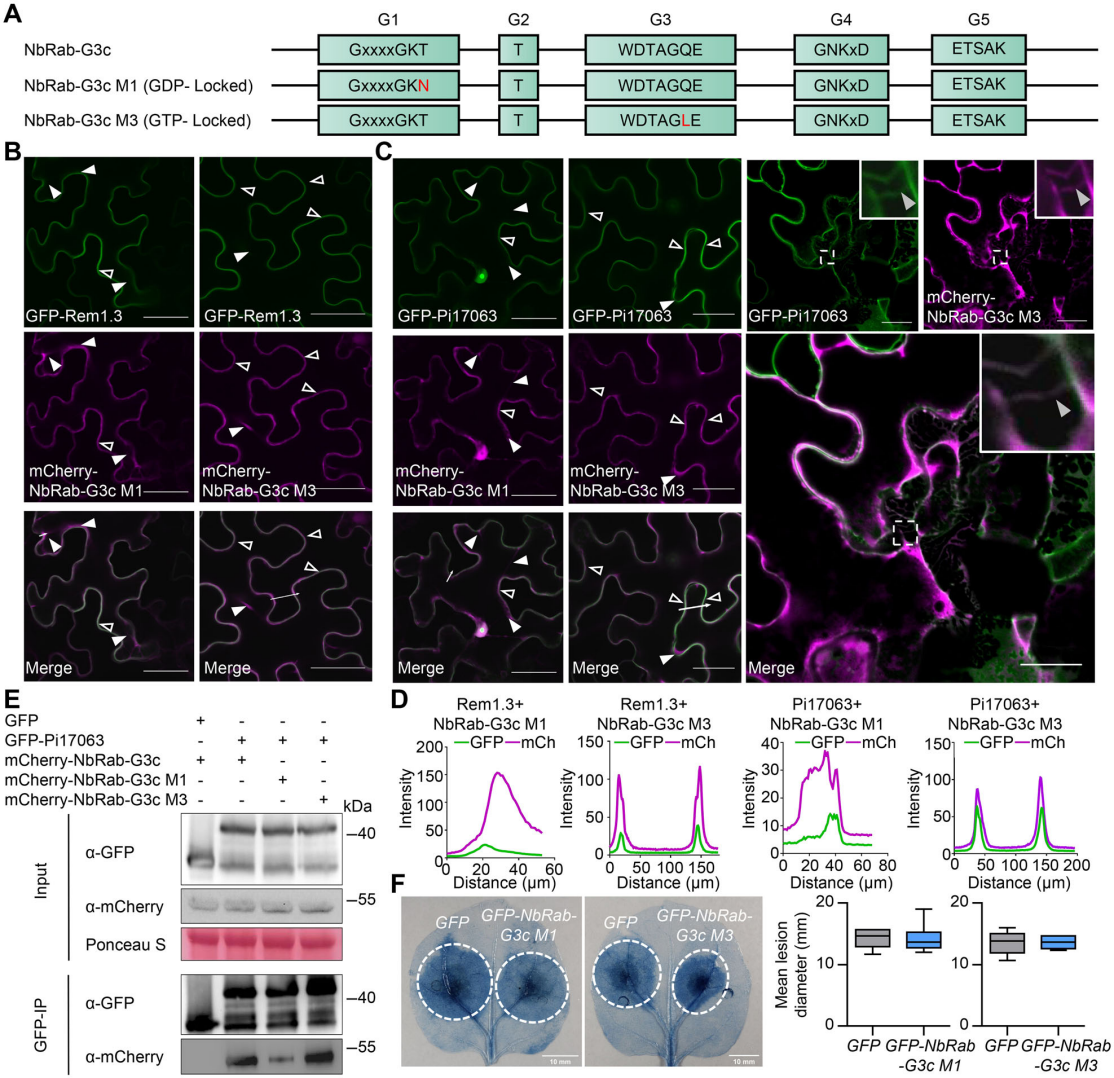
GTPase activity of NbRab‐G3c is indispensable for its localization and immune function **(A)** Schematic representation of NbRab‐G3c GTPase mutant constructs. Red residues indicate mutation sites. **(B)** PM localization of NbRab‐G3c mutants. **(C)** Subcellular co‐localization of NbRab‐G3c mutants with Pi17063. **(D)** Fluorescence intensity was quantified along the transects (white line) using ImageJ software. **(E)** Co‐IP assays using GFP‐trap beads. Total proteins were extracted from *N. benthamiana* leaves at 3 dpi. Co‐precipitation was detected via western blotting. Ponceau S staining indicates protein samples loaded. **(F)** Immune function of NbRab‐G3c mutants. *GFP* was used as the control. Lesion images were stained with trypan blue, and dotted white circles indicate lesion areas. Data are obtained from at least 10 leaves. The upper quartile, median, and lower quartile are shown in each box plot, while the bars outside the box indicate the maximum and minimum values. Confocal images information: Scale bars = 40 μm. Fluorescence intensity was quantified in the transect (white line) using ImageJ software. Examples of PM, cytoplasmic and hechtian strand localization are highlighted with open, white‐filled and gray‐filled triangles, respectively.

To further analyze the mechanism by which Pi17063 exerted GAP activity to suppress plant immunity, we determined the immune function of NbRab‐G3c in different states. We created two NbRab‐G3c GTPase mutant constructs, *NbRab‐G3cM1* (GDP‐Locked) and *NbRab‐G3cM3* (GTP‐Locked) based on a previous report (Nielsen, 2020) (Figure 5A). Their ability to hydrolyze GTP was tested using a Plant GTP ELISA Kit (MM‐3590802), with results showing completely lost GTPase activity for these two mutants (Figure S13A, B), which indicated that the designed mutants could be used for subsequent analysis.

We then performed a co‐localization analysis of NbRab‐G3cM1 or NbRab‐G3cM3 with Rem1.3. Consistent with the characteristics of small GTPases, NbRab‐G3cM1 mainly localized in the cytoplasm, while NbRab‐G3cM3 largely localized on PM, with only a little cytoplasmic localization (Figures 5B, D, S13C). Subcellular co‐localization of GFP‐Pi17063 and mCherry‐NbRab‐G3c mutant variants was further examined. mCherry‐NbRab‐G3cM3 mostly co‐localized with GFP‐Pi17063 on PM and hechtian strands (with plasmolysis treatment), while mCherry‐NbRab‐G3cM1 was more concentrated in the cytoplasm, with a small amount co‐localized with GFP‐Pi17063 on the PM (Figure 5C, D). The interaction between NbRab‐G3c mutant proteins and Pi17063 was further tested by co‐IP and ITC assays, with results showing that Pi17063 exhibited a stronger binding affinity to NbRab‐G3c M3 compared with that of NbRab‐G3c M1 (Figures 5E, S14). The preference for binding to NbRab‐G3c in the active state was consistent with the GAP activity of Pi17063.

Last, we tested the immune functions of these mutants in *N. benthamiana*. There were no detectable differences in either lesion size or *P. infestans* colonization between the mutants and the *GFP* control (Figure 5F, S13D, E). Consistent with this, two NbRab‐G3c GTPase mutants had lost the ability to promote both PTI‐associated *WRKY7* expression (Figure S13F) and ROS bursts triggered by flg22 (Figure S13G). These results suggested that positive regulation of plant defense responses mediated by NbRab‐G3c requires its full ability to convert between the active and inactive states.

### NbGYP is an endogenous GAP on NbRab‐G3c and regulates plant immunity

**Figure 6:**
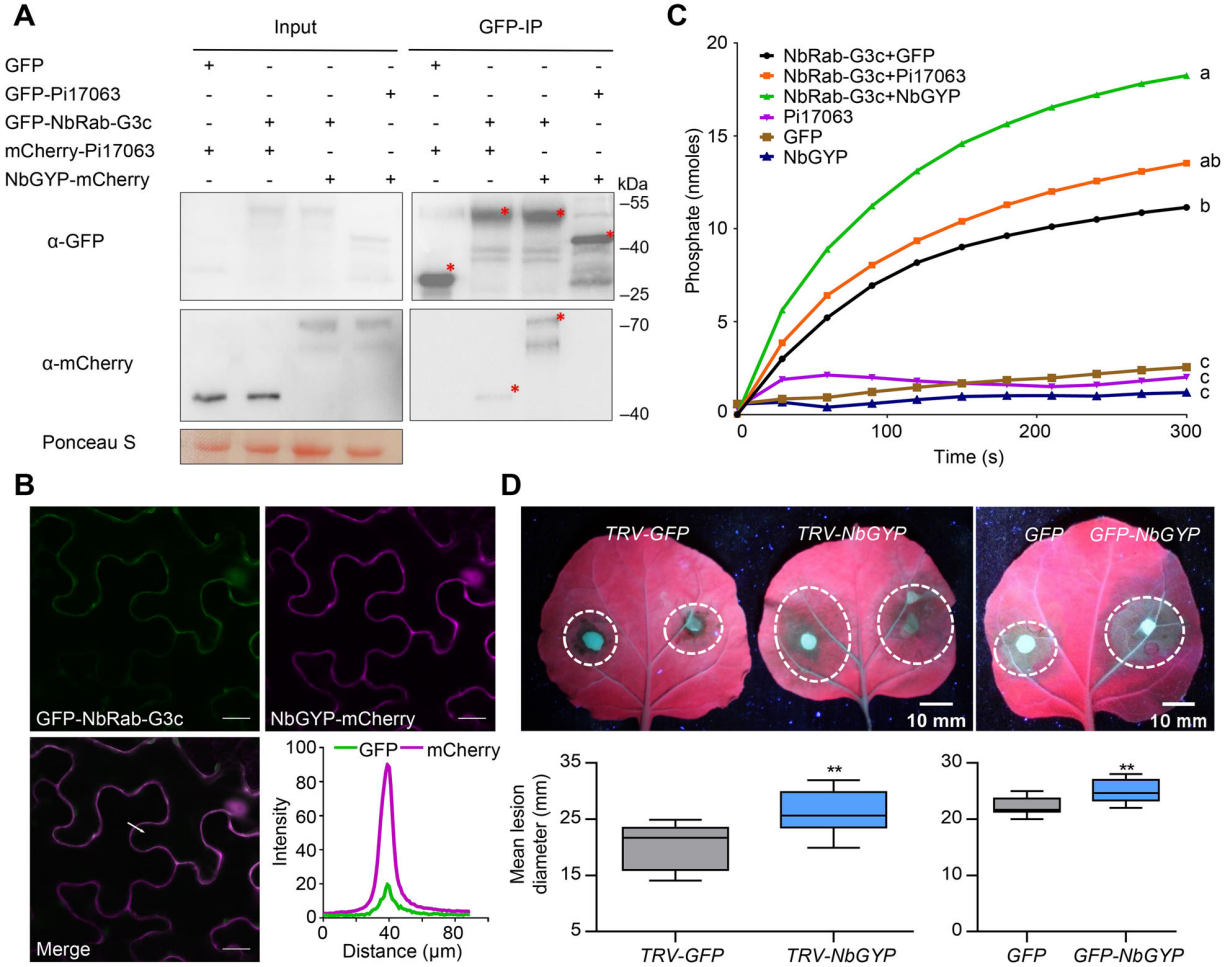
Proper balanced active and inactive states of NbRab‐G3c are crucial for contribution to plant immunity **(A)** Co‐IP assays were used to assess the interaction of NbGYP with NbRab‐G3c and Pi17063. Co‐precipitation was detected via western blotting. Asterisks indicate bands of the expected sizes. **(B)** Co‐localization of GFP‐NbRab‐G3c and NbGYP‐mCherry. Scale bars = 20 μm. Fluorescence intensity analysis in the transect (white line). **(C)** *In vitro* GAP activity of NbGYP. The data at 300 s of reaction were subjected to Tukey's multiple comparisons test; different letters indicate significant differences (*P* < 0.05). **(D)** Immune function of NbGYP. *N. benthamiana* leaves transiently overexpressing *GFP‐NbGYP* or *GFP* (6 biologically independent samples), or expressing silencing constructs of *GFP* and or *NbGYP* (12 biologically independent samples) were challenged with *P. infestans* zoospores. Lesion images were taken under UV light. Dotted white circles indicate lesion areas. The upper quartile, median, and lower quartile are shown in each box plot, while the bars outside the box indicate the maximum and minimum values. **P < 0.01 (Student's *t*‐test).

To further confirm the importance of full NbRab‐G3c switching for its positive regulation of plant resistance, we searched for *N. benthamiana* proteins annotated as GAPs using the BLAST tool in the Solanaceae Genomics Network (https://solgenomics.net/tools/blast/). This yielded a predicted endogenous GAP, NbGYP (Niben101Scf08280g01020.1). Co‐IP assays confirmed the interaction between NbGYP and NbRab‐G3c (Figure 6A). Confocal observation also showed obvious co‐localization of NbGYP‐mCherry and GFP‐NbRab‐G3c on PM (Figure 6B). Furthermore, recombinant NbGYP protein was prepared (Figure S11C) and co‐incubated with recombinant NbRab‐G3c protein to confirm the ability of NbGYP to promote GTPase activity. Indeed, the ability of NbRab‐G3c to hydrolyze GTP was significantly enhanced by co‐incubation with NbGYP (Figure 6C). Taken together, these results indicated that NbGYP was an endogenous GAP.

To further examine the effect of NbGYP on immune function, we prepared *NbGYP*‐silenced *N. benthamiana* plants using a TRV‐based VIGS method. RT‐qPCR results showed a decrease of more than 70% in *NbGYP* transcripts in *TRV‐NbGYP* plants (Figure S15A) without significant effects on growth phenotypes (Figure S15B). *TRV‐NbGYP* plants showed significantly larger lesion diameters (Figure 6D) and increased pathogen colonization (Figure S15C) compared with the control in infection assays with *P. infestans* zoospores. Expression of PTI‐related *WRKY7* was also down‐regulated in *TRV‐NbGYP* leaves after INF1 treatment (Figure S15D). This suggested that the ability of NbRab‐G3c (and probably other NbRab‐G3 proteins) to switch between states was suppressed in the *NbGYP*‐silenced leaves, degrading overall immune function. To further confirm this, *NbGYP* was transiently expressed in *N. benthamiana* leaves, followed by inoculation with *P. infestans* zoospores. Transient overexpression of *NbGYP* rendered plants more susceptible to *P. infestans* infection (Figures 6D, S15C, E). These results suggested that both reduced and elevated GAP expression interfered with the ability of NbRab‐G3c (and probably other NbRab‐G3s) to mediate resistance to *P. infestans*. This was presumably due to the suppression of the complete conversion cycle between the active and the inactive states of the NbRab‐G3s, which was required for them to function as positive immune regulators.

In addition, the results of co‐IP assays also showed that Pi17063 did not interact with NbGYP (Figure 6A), indicating that the direct regulation of NbRab‐G3c is associated with the virulence function of Pi17063, which is independent of NbGYP. ITC assay also showed a slightly weaker interaction of Pi17063 and NbRab‐G3c than that of NbGYP and NbRab‐G3c (Figure S16), indicating that Pi17063 does not compete with NbGYP to bind to NbRab‐G3c.

## DISCUSSION

Plant Rab‐G GTPases were reported to be localized to the vacuolar membrane and to participate in endomembrane and vesicle trafficking (Bozkurt et al., 2015). However, our results showed that NbRab‐G3c GTPase was localized with Pi17063 on the PM as well as in the cytoplasm (Figure 3). This may be due to the functional differentiation of Rab GTPases among different plant species. Small GTPases with PM localization are usually involved in the recycling and transportation of specific receptors and defense‐related components to the cell membrane (Choi et al., 2013; Tomczynska et al., 2018). Our results further revealed that different members of the NbRab‐G3 subfamily GTPases showed cytoplasmic localization in addition to co‐localization with Pi17063 on the PM (Figures 3, S8), and acted as positive regulators of plant immunity (Figures S9, S10). In *A. thaliana*, Rab‐G subfamily GTPases with PM localization are involved in various cellular processes such as vacuole biogenesis, autophagy and vesicle trafficking (Rojo et al., 2001; Kwon et al., 2010). It remains to be determined exactly which sets of the immune functions of all NbRab‐G3 proteins against diverse pathogens are shared and distinct, and the extent to which Pi17063 interferes with each protein.

Targeting and regulating Rab GTPase functions is one of the key strategies of effectors to suppress host immunity (Tomczynska et al., 2018; Li et al., 2022). Our results showed that Pi17063 preferentially interacted with NbRab‐G3c in the active state and functioned as a GAP (Figure 4A), switching the host Rab‐G3 GTPases from the active (GTP‐bound) to the inactive (GDP‐bound) state. Previous studies found that Rab GTPase activities can be regulated by effectors from *L. pneumophila* (Mukherjee et al., 2011; Tan et al., 2011), *S. flexneri* (Dong et al., 2012) and *Salmonella* (Spanò et al., 2016) in mammalian cells. However, a similar biochemical mechanism has not been reported in fungal or oomycete pathogens. Here we report that Pi17063 functions as a GAP of PM‐localized NbRab‐G3 subfamily GTPases. Pi17063 is a small secreted protein, lacking typical GAP domains, and its catalytic activity and virulence function rely on the conserved arginine residues (Figure 4), suggesting a new mechanism by which Pi17063 promotes the GTPase activity of Rab‐G3 proteins in a way different from that of endogenous GAPs or bacterial effectors. Interestingly, effectors with GAP activity are often accompanied by effectors with GEF activity (Tan et al., 2011). Further genomic and biochemical analysis will be necessary to determine whether any *P. infestans* effectors can promote NbRab‐G3 GTPase dissociation from GDP and binding with new GTP.

The different states of small GTPases are crucial for their function. In *A. thaliana*, the *Pseudomonas syringae* effector AvrPto targets the Rab GTPase AtRab‐E1d (Bogdanove and Martin, 2000). Overexpression of the active form of AtRab‐E1d (AtRab‐E1d‐Q74L) increases host resistance to *P. syringae*, but the inactive form of AtRab‐E1d does not play any regulatory role in plant immunity (Speth et al., 2009). We here found that both GDP‐ and GTP‐locked NbRab‐G3c lacked plant immune regulatory activity (Figure 5). This indicated that NbRab‐G3c function in plant immunity may not rely solely on its ability to bind and regulate downstream proteins and molecular compounds, but also on the homeostasis of the two states. This was supported by results from silencing and overexpressing the endogenous GAP gene of NbRab‐G3c, *NbGYP*, both of which inhibited plant immunity (Figure 6). Rab‐G subfamily members are involved in vesicle and endomembrane trafficking, which are essential for cellular processes and are strictly regulated by GEFs and GAPs (Takai et al., 2001; Nielsen et al., 2008). In addition, the regulation of downstream signaling effectors by small GTPases is highly dependent on their GTP‐bound active state, and GTP can typically be slowly hydrolyzed in the absence of GAPs (Lamber et al., 2019). Therefore, the number of endogenous GAPs is relatively fewer compared with the number of GEFs in plants. Pi17063 did not show significantly stronger interaction with NbRab‐G3c than NbGYP (Figure S16), possibly because Pi17063 and NbGYP showed similar immune and biochemical functions. Without competing with NbGYP, the presence of Pi17063 greatly accelerates the switch of intracellular NbRab‐G3 GTPases from the active to the inactive state, disrupting the balance between the two states and enhancing plant susceptibility.

**Figure 7:**
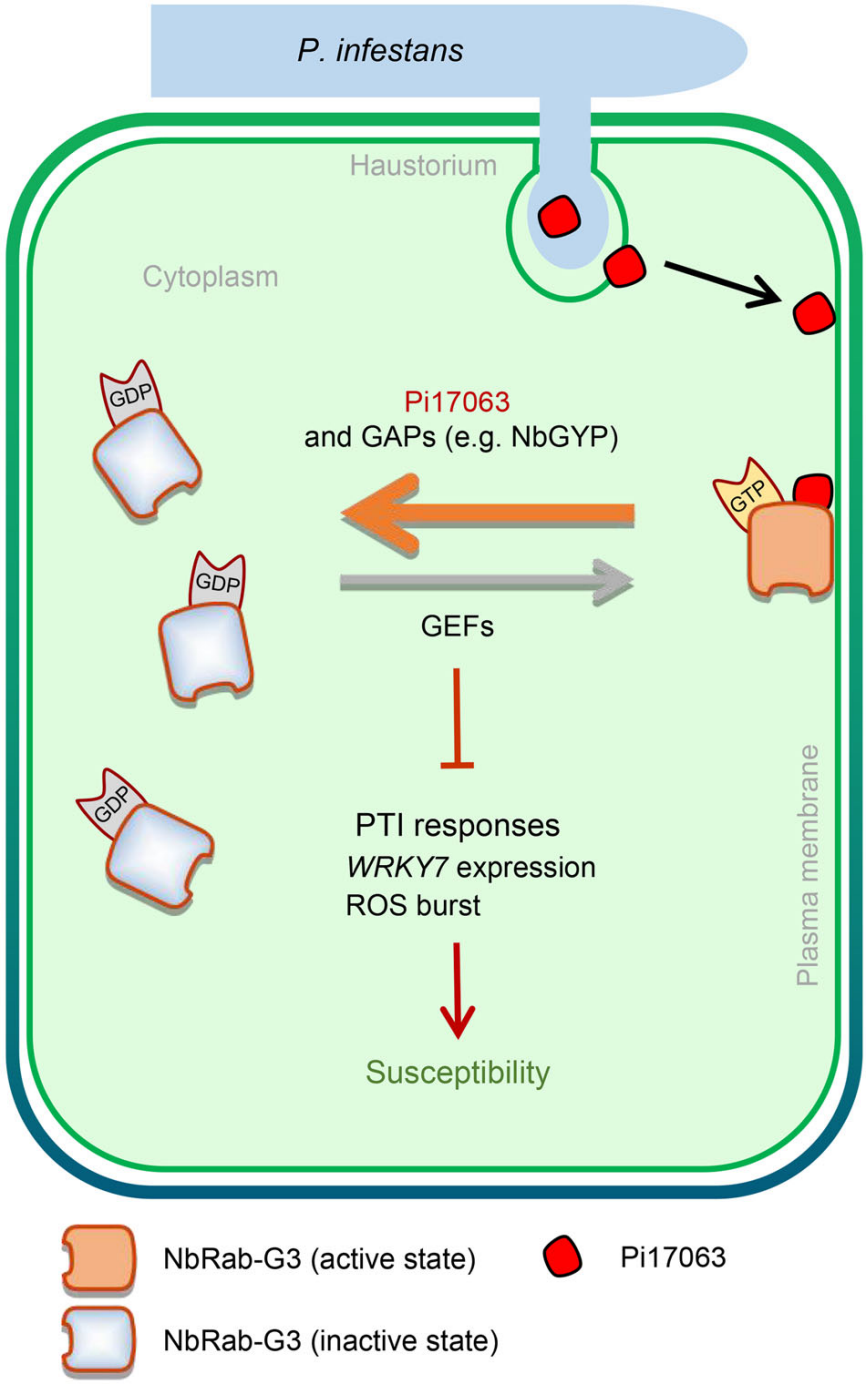
A model for suppression of plasma membrane‐localized NbRab‐G3c‐mediated plant immunity by the late blight effector Pi17063 NbRab‐G3c protein exists in the active (GTP‐bound, plasma membrane‐localized) and inactive (GDP‐bound, cytoplasm‐localized) states in plants; their switch is regulated by endogenous GAPs (e.g., NbGYP) and GEFs. Strict regulation of the NbRab‐G3c state is essential for its role in plant immunity and PTI responses. The host PM‐localized *P. infestans* effector Pi17063 functions as a GAP to promote NbRab‐G3c changing from the active state to the inactive state, leading to disruption of the NbRab‐G3c switch cycle and enhanced plant susceptibility to *P. infestans*. Other NbRab‐G3 family members are likely to follow the same model.

In summary, the core late blight effector Pi17063 targets host plant PM‐localized NbRab‐G3 GTPases. The four tested members of the NbRab‐G3 subfamily positively regulate plant immunity. Pi17063 functions as a GAP and promotes the GTPase activity of NbRab‐G3 proteins, accelerating their switch from the active GTP‐bound to the inactive GDP‐bound state. NbRab‐G3c mutants locked in either the active or inactive state lost their immune regulatory function. Thus, Pi17063 disrupted NbRab‐G3 state switching, enhancing plant susceptibility to *P. infestans* (Figure 7). Our findings illustrate an underlying biochemical mechanism of an oomycete effector in targeting plant PM‐localized small GTPases, providing insight into plant susceptibility triggered by a late blight effector.

## MATERIALS AND METHODS

### Plant growth conditions

*N. benthamiana* and *S. tuberosum* plants were grown in the growth chamber with 5000 Lx (LED lamps, OUTRACE, OU016‐U4B) and a 13/11 h light/dark cycle at 25°C.

### Bacteria, *P. infestans* and their growth conditions

*Escherichia coli* DH10B strain was used for plasmid construction, BL21 (DE3) strain was used for recombinant protein preparation, *Agrobacterium tumefaciens* GV3101 strain was used for transient expression and VIGS assays. All the above bacteria were cultured in Luria–Bertani (LB) medium (10 g/L NaCl, 10 g/L peptone and 5 g/L yeast extract). *E. coli* was cultured at 37°C and *A. tumefaciens* was cultured at 28°C.

*P. infestans* isolate 88069 was used for the infection assay. It was grown in the temperature incubator (16°C) on Rye‐sucrose agar medium (RSA) (60 g/L Rye, 10 g/L sucrose and 8 g/L agar) plates.

### Plasmid construction

All the primers used in this study are listed in Table S2. For LCI assays, *Pi17063* (encoding mature protein without signal peptide) was cloned into the *pCAMBIA‐nLuc* vector (Zhou et al., 2018) using *Bam*HI and *Sal*I sites to form plasmid *Pi17063‐nLuc*. *NbRab‐G3* subfamily genes were amplified from cDNA generated from *N. benthamiana* and cloned into the *pCAMBIA‐cLuc* vector (Zhou et al., 2018) using *Bam*HI and *Sal*I sites to form the corresponding *cLuc‐NbRabs* plasmids. For VIGS assays, 200–300‐bp fragments of *NbRab‐G3c* and *NbGYP* were selected using the VIGS tool (https://vigs.solgenomics.net/), which were amplified from the cDNA of *N. benthamiana*. The fragments of *NbRab‐G3c* and *NbGYP* were cloned into the *pTRV2* vector (Senthil‐Kumar and Mysore, 2014) using *Eco*RI and *Xba*I. For overexpression, *NbRab* genes and *NbGYP* were inserted into *pART27‐mCherry* between the *Eco*RI and *Xho*I sites. *Pi17063*, *Pi17063 M*, *NbRab* genes and *NbRab‐G3c M* were cloned into *pART27‐GFP* between the *Eco*RI and *Xba*I sites. Both of them were under the control of the *CaMV 35S* promoter (Gleave, 1992; Wesley et al., 2001). Two new GATEWAY vectors (*pDEST‐VYNE(R)* ^*GW*^ and *pDEST‐VYCE(R)* ^*GW*^) were used for BiFC assays (Gehl et al., 2009), and *NbRab*‐G3c and *Pi17063* were cloned into them between the *Spe*II and *Xho*I sites respectively. For expression and purification of recombinant proteins, *Pi17063*, *Pi17063 M*, *NbRab‐G3c* and *NbGYP* were cloned into the *pET32a* vector (Zhang et al., 2024) using the *Nco*I and *Xho*I sites to obtain *PET‐Pi17063*, *PET‐Pi17063 M*, *PET‐NbRab‐G3c* and *PET‐NbGYP* plasmids for prokaryotic recombinant protein expression.

### PAMP treatments and ROS production

Leaves from *TRV‐NbRab‐G3* and *TRV‐GFP N. benthamiana* plants, or *Pi17063*, *NbRab‐G3* and *GFP* overexpression plants were infiltrated with 10 μM Flg22 or 10 μM INF1. The ROS burst was measured using a luminol‐based assay as previously reported (Yang et al., 2022).

### Pathogen inoculation analysis

Six‐week‐old *N. benthamiana* plants were used for pathogen inoculation analysis. Zoospores of *P. infestans* isolate 88069 suspensions were prepared with cold distilled water (4°C). Approximately 1,000 zoospores were inoculated on each spot of *N. benthamiana* leaves. *P. infestans*‐inoculated leaves were kept in the dark for 7 d before lesion diameters were measured. Each inoculation test had approximately 30 biological replicates from three independent replicates. For measurement of the relative biomass of *P. infestans* within *N. benthamiana* leaves, DNA was extracted from the lesion areas of at least six *N. benthamiana* leaves and pooled. Quantitative PCR assays of genomic DNA were used employing primers specific for the *PiUBC* (*PITG_08327*) and *NbActin* genes. Statistical analysis results are listed in Table S3.

### LC–MS/MS analysis

Total protein was extracted from *N. benthamiana* leaves using RIP protein extraction buffer (50 mM Tris‐HCl pH 7.4, 150 mM NaCl, 1% Triton‐X‐100, 1% sodium deoxycholate, 0.1% SDS, 3 mM NaF, 1 mM sodium orthovanadate, 1 mM EDTA, 2% PVPP, 1 mM DTT and 1 mM PMSF) with a protease inhibitor cocktail (Sigma, Missouri, USA). GFP‐trap agarose beads (Chromotek, New York, USA) were used for immunoprecipitation according to the manufacturer's protocol. Protein samples were digested into short peptides using trypsin hydrolysis and then analyzed by liquid chromatography‐tandem mass spectrometry (Triple Quad 5500 + QTRAP Ready, AB SCIEX, Queenstown, Singapore).

### Confocal microscopy imaging

Confocal imaging was performed using an Olympus FV3000 confocal microscope (Tokyo, Japan) with HCX APOL ×10/0.4, ×20/0.75 and ×40/0.95 lenses. Respective fusion constructs were co‐expressed via agroinfiltration in *N. benthamiana* leaves 3 d before confocal observation. For PM or nucleus staining, 10 μM FM4‐64 or 10 μM DAPI was infiltrated into *N. benthamiana* leaves for 15 min before observation. For observation of hechtian strands, 2 M NaCl was used to induce plasmolysis. DAPI was excited at 340 nm and detected at 460–490 nm. GFP was excited at 488 nm and detected at 500–540 nm. For mCherry or FM4‐64 observation, fluorescence was excited at 559 nm and detected at 580–630 nm.

### Co‐immunoprecipitation and western blot analysis

Agroinfiltrated *N. benthamiana* leaves were harvested at 3 days post infiltration (dpi) and total protein was extracted using RIP protein extraction buffer. Total protein extract was incubated with 20 μL GFP‐trap agarose beads (Chromotek, New York, USA) at 4°C for 4 h. The beads were then washed with IP buffer (10% glycerol, 25 mM Tris‐HCl pH 7.5, 1 mM EDTA, 150 mM NaCl) four to six times. The resuspended beads were oscillated using a vortex meter to dissociate the complexes.

PM proteins were extracted using the Minute^TM^ Plasma Membrane Protein Isolation Kit (SM‐005‐P, BioVision, California, USA) following the manufacturer's protocol. Western blot assays were performed as previously reported (Du et al., 2021). Primary antibodies: rabbit anti‐GFP (AE011, ABclonal, Hubei, China) and mouse anti‐mCherry (AE002, ABclonal, Hubei, China) at 1:2000 dilution. Horseradish peroxidase‐conjugated secondary antibodies: anti‐rabbit (AS014, ABclonal, Hubei, China) and anti‐mouse (AS003, ABclonal, Hubei, China) at 1:2000 dilution.

### Agroinfiltration and VIGS assays

*Agrobacterium tumefaciens* strain AGL1 transformed with plasmids described above were grown in LB medium with appropriate antibiotics at 28°C for 2 d. After centrifugation and resuspension in 2‐(*N*‐morpholino) ethanesulfonic acid (MES) infiltration medium (200 μM acetosyringone, 10 mM MES pH 5.6, 10 mM MgCl_2_·6H_2_O), bacterial suspensions were adjusted to an OD_600_ of 0.1 for confocal microscopy and infection assays, 0.2 for LCI assays and 0.3 for co‐IP assays. For co‐expression, the bacterial cultures transformed with different vectors were mixed in a 1:1 ratio and their concentrations were adjusted as described above.

For VIGS, *A. tumefaciens* strains GV3101 carrying *TRV1*, or *TRV2‐GFP*, *TRV2‐PDS*, *TRV2‐NbRab‐G3* and/or *TRV2‐NbGYP* were mixed in a 1:1 ratio and their concentrations were adjusted to an OD_600_ of 0.2. The bacterial cultures were infiltrated into the fifth and sixth leaves of 3‐week‐old *N. benthamiana* plants.

### Effector elicitor activity assays

Agroinfiltration assay in potato was performed as described previously (Elnahal et al., 2020). Briefly, *A. tumefaciens* strain AGL1 cell suspensions were adjusted to an OD_600_ of 0.4 and infiltrated into the leaves of 9‐week‐old potatoes. For the protein infiltration assay, the purified recombinant effector proteins were diluted with ddH_2_O to a concentration of 50 μM and infiltrated directly into 9‐week‐old potato leaves. HR was monitored and photographed at 4 d post agroinfiltration and 2 d post protein infiltration.

### Gene transcript level assays

Total RNA was extracted using TRIzol reagent (Invitrogen, California, USA). Approximately 1 μg RNA was used to synthesize cDNA by PrimeScript^TM^ RT reagent Kit (TaKaRa, Liaoning, China) following the manufacturer's protocol. The cDNA was diluted 10‐fold and then 5 μL of the diluent was used as the template for each quantitative reverse transcription PCR assay (qRT‐PCR). The qRT‐PCR was performed using a SYBR Green master mix (Roche, California, USA) and specific primers in an iQ7 Real‐Time Cycler (Life Technologies, California, USA). *NbActin* was used as a reference gene in *N. benthamiana* for normalization. *PiUBC* was used as a reference gene in *P. infestans* for normalization. Gene relative transcript levels were quantified via the 2^−ΔΔCt^ method. All primers are listed in Table S2. Statistical analysis results are listed in Table S3.

### Recombinant protein purification and *in vitro* GTPase activity analysis

The constructs of *PET‐Pi17063*, *PET‐Pi17063 M*, *PET‐NbRab‐G3c*, *PET‐NbRab‐G3c M*, and *PET‐NbGYP* were transformed into *Escherichia coli* strain BL21‐DE3 and cultivated in LB broth until the OD_600_ reached 0.6. Bacteria were then induced with 1 mM IPTG for 12 h at 18°C. The bacteria were then collected by centrifugation and resuspended in lysis buffer (20 mM pH 7.5 Tris‐HCl and 40 mM NaCl). The ultrasonic cell disruptor was used for breaking *E. coli* cells, and the proteins were recovered from the supernatant using affinity chromatography and Ni NTA Beads 6FF (Smart‐Lifesciences, Jiangsu, China). The GTPase activity of NbRab‐G3c was quantified using an ATPase/GTPase ELIPA Biochem Kit (Cytoskeleton, Washington, USA) following the manufacturer's protocol. The ability of NbRab‐G3c and its mutants to hydrolyze GTP was quantified by Plant GTP ELISA Kit (MEIMIAN, Jiangsu, China) following the manufacturer's protocol. Statistical analysis results are listed in Table S3.

### ITC assay

The purified recombinant NbGYP, NbRab‐G3c, NbRab‐G3cM and Pi17063 proteins were vacuum degassed before the test. Pi17063 or NbGYP was added to the sample pool, titrated with NbRab‐G3c at constant temperature, and the heat change was recorded by Nano‐ITC (Waters Corporation, Massachusetts, USA). Data were analyzed by ITCRun software.

### Statistical analysis

Raw data from the infection assay (lesion diameters), RT‐qPCR (relative transcript level and pathogen biomass), and GTPase content test assays performed in this study were all analyzed using GraphPad Prism with Student's *t*‐test. Raw data from the GTPase activity test were analyzed using Data Processing Station (DPS) with Tukey's multiple comparisons test.

Statistical methods, differences between means, 95% confidence intervals and *P*‐values are listed in Table S3. “*n*” represents the number of bio‐repeats, the value of which are all listed in figure legends. Error bars are defined in figure legends. *P < 0.05, **P < 0.01, ***P < 0.001.

### Image processing and data analysis

Fluorescence intensity analysis in co‐localization sites was performed using ImageJ software. Confocal images with a single channel were stacked in ImageJ software, and the gray values of co‐localization sites were derived. The plot file data were prepared as a single graph in GraphPad Prism.

## CONFLICTS OF INTEREST

The authors declare no conflict of interest.

## AUTHOR CONTRIBUTIONS

S.L., X.L., and X.X. performed most of the research and S.L. drafted the manuscript. L.D. carried out recombinant protein purification experiments. J.L. performed the effector elicitor activity test. X.Z. and Z.L. carried out the bioinformatics analysis. Y.L. performed ITC assays. T.Y., Y.H., Y.W., and X.C. constructed most of the plasmids and performed some infection assays. S.L., Y.M., and W.S. designed the experiments, and revised the manuscript. W.S. supervised the study. All authors read and approved of its content.

## Supporting information

Additional Supporting Information may be found online in the supporting information tab for this article: http://onlinelibrary.wiley.com/doi/10.1111/jipb.13920/suppinfo

**Figure S1.** Pi17063 is host PM‐localized
**Figure S2.** Nuclear‐localized Pi17063 is abolished to inhibit plant PTI response
**Figure S3.** Overexpression of *mCherry‐Pi17063* renders *N. benthamiana* more susceptible to *P. infestans*
**Figure S4.** Pi17063 co‐localizes with NbRab‐G3c on PM
**Figure S5.** Pi17063 interacts with NbRab‐G3c on PM
**Figure S6.** Pi17063 co‐localizes with, but cannot interact with, NbRab‐A–NbRab‐F subfamily GTPases
**Figure S7.** Pi17063 specifically interacts with the NbRab‐G3 subfamily of GTPases
**Figure S8.** NbRab‐G3 subfamily proteins localize and co‐localize with Pi17063 on PM
**Figure S9.** NbRab‐G3 subfamily members NbRab‐G3f1, NbRab‐G3f2, and NbRab‐G3a positively regulate plant immunity
**Figure S10.** *NbRab‐G3* genes positively regulate plant immunity and PTI responses
**Figure S11.** Integrity and functional confirmation of recombinant proteins produced in *E. coli*
**Figure S12.** Pi17063M2 retains host PM localization and promotes *P. infestans* colonization in *N. benthamiana*
**Figure S13.** NbRab‐G3cM1 and NbRab‐G3cM3 mainly localize on cytoplasm and PM, respectively, and both lose the ability to regulate plant immunity and PTI responses
**Figure S14.** Pi17063 preferentially interacts with the GTP‐bound NbRab‐G3c
**Figure S15.** Both silencing and overexpression of *NbGYP* render *N. benthamiana* more susceptible to *P. infestans*
**Figure S16.** Interaction intensity of Pi17063 with NbRab‐G3c is slightly weaker than NbGYP
**Table S1.** Results of LC–MS/MS
**Table S2.** Primers used in this study
**Table S3.** Statistical analysis tables

## Data Availability

*Pi17063* (PITG_17063 in the NCBI database) has accession number XM_002897216.1. Sequence data for genes cloned from *N. benthamiana* can be found in the Solanaceae Genomics Network (https://solgenomics.net): *NbRab‐G3c* (Niben101Scf01374g03034.1), *NbRab‐G3a* (Niben101Scf08526g01012.1), *NbRab‐G3f1* (Niben101Scf04778g00002.1), *NbRab‐G3f2* (Niben101Scf06726g00033.1), *NbRab‐A5e* (Niben101Scf00270g12012.1), *NbRab‐B1b* (Niben101Scf01517g08034.1), *NbRab‐E1c1* (Niben101Scf00684g00002.1), *NbRab‐D1* (Niben101Scf05032g00006.1), *NbRab‐E1c2* (Niben101Scf09596g00001.1), *NbRab‐F2a* (Niben101Scf00271g01020.1), *NbGYP* (Niben101Scf08280g01020.1).
