# Supplementary material for: Late blight pathogen targets host Rab‐G3 GTPases with an atypical GTPase‐activating protein
Source: J Integr Plant Biol. 2025 May 7;67(8):2135–50. doi: 10.1111/jipb.13920 (PMC12315501; doi:10.1111/jipb.13920)
Supplement: Supplementary file 1 — Figure S1. Pi17063 is host PM‐localized Figure S2. Nuclear‐localized Pi17063 is abolished to inhibit plant PTI response Figure S3. Overexpression of mCherry‐Pi17063 renders N. benthamiana more susceptible to P. infestans Figure S4. Pi17063 co‐localizes with NbRab‐G3c on PM Figure S5. Pi17063 interacts with NbRab‐G3c on PM Figure S6. Pi17063 co‐localizes with, but cannot interact with, NbRab‐A–NbRab‐F subfamily GTPases Figure S7. Pi17063 specifically interacts with the NbRab‐G3 subfamily of GTPases Figure S8. NbRab‐G3 subfamily proteins localize and co‐localize with Pi17063 on PM Figure S9. NbRab‐G3 subfamily members NbRab‐G3f1, NbRab‐G3f2, and NbRab‐G3a positively regulate plant immunity Figure S10. NbRab‐G3 genes positively regulate plant immunity and PTI responses Figure S11. Integrity and functional confirmation of recombinant proteins produced in E. coli Figure S12. Pi17063M2 retains host PM localization and promotes P. infestans colonization in N. benthamiana Figure S13. NbRab‐G3cM1 and NbRab‐G3cM3 mainly localize on cytoplasm and PM, respectively, and both lose the ability to regulate plant immunity and PTI responses Figure S14. Pi17063 preferentially interacts with the GTP‐bound NbRab‐G3c Figure S15. Both silencing and overexpression of NbGYP render N. benthamiana more susceptible to P. infestans Figure S16. Interaction intensity of Pi17063 with NbRab‐G3c is slightly weaker than NbGYP Table S1. Results of LC–MS/MS Table S2. Primers used in this study Table S3. Statistical analysis tables [file JIPB-67-2135-s001.docx]

**SUPPORTING INFORMATION**

**Running Title: Pi17063 activates host Rab-G3 GTPases as a GAP**

**Late blight pathogen targets host Rab-G3 GTPases with an atypical GTPase activating protein**

**Song Liu^1^, Liwen Ding^1^, Xiong Liu^1^, Xiaoxi Xing****^1^, Jinyang Li^1^, Tiantian Yan^1^,Yuli Huang^1^, Yuan Liu^1^, Yisa Wang^1^, Xia Zhang^1^, Zeming Liu^1^, Xiyu Cao^1^, Yuling Meng^1^ and Weixing Shan^1*^**

^1^State Key Laboratory of Crop Stress Resistance and High-Efficiency Production and College of Agronomy, Northwest A&F University, Yangling, Shaanxi 712100, China.

*Correspondence: Weixing Shan (wxshan@nwafu.edu.cn)

**
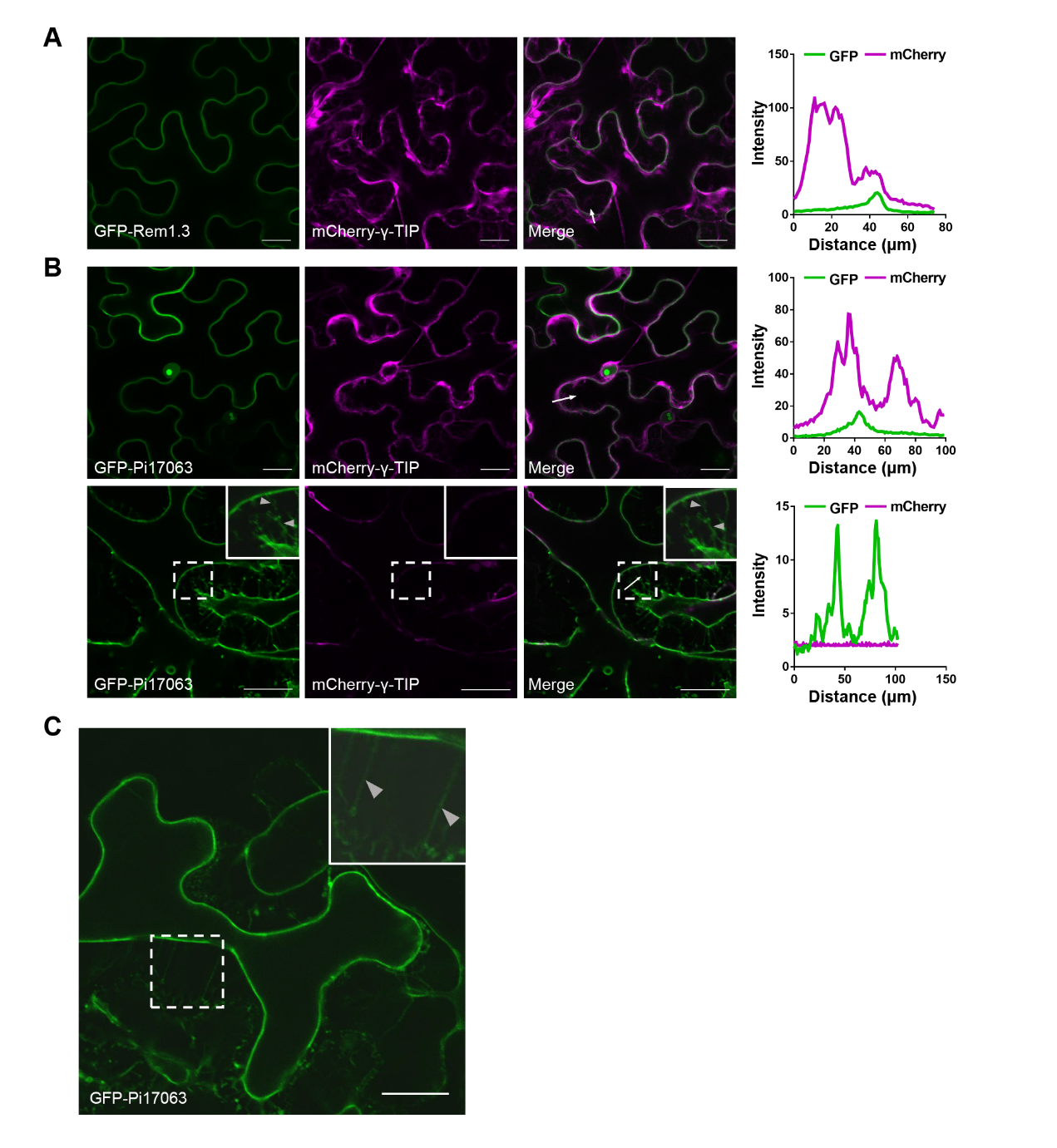
**

**Figure S1 Pi17063 is host PM-localized.**

**(A)** localization of mCherry-γ-TIP relative to GFP-Rem1.3. **(B)** localization of Pi17063 relative to vacuolar membranes. **(C)** hechtian strands of GFP-Pi17063. Confocal images information: Scale bars = 20 μm. Fluorescence intensity was quantified along the transects (white line) with ImageJ. The white dashed squares indicate local zoom-in insets. Examples of hechtian strands are highlighted with gray filled triangles.


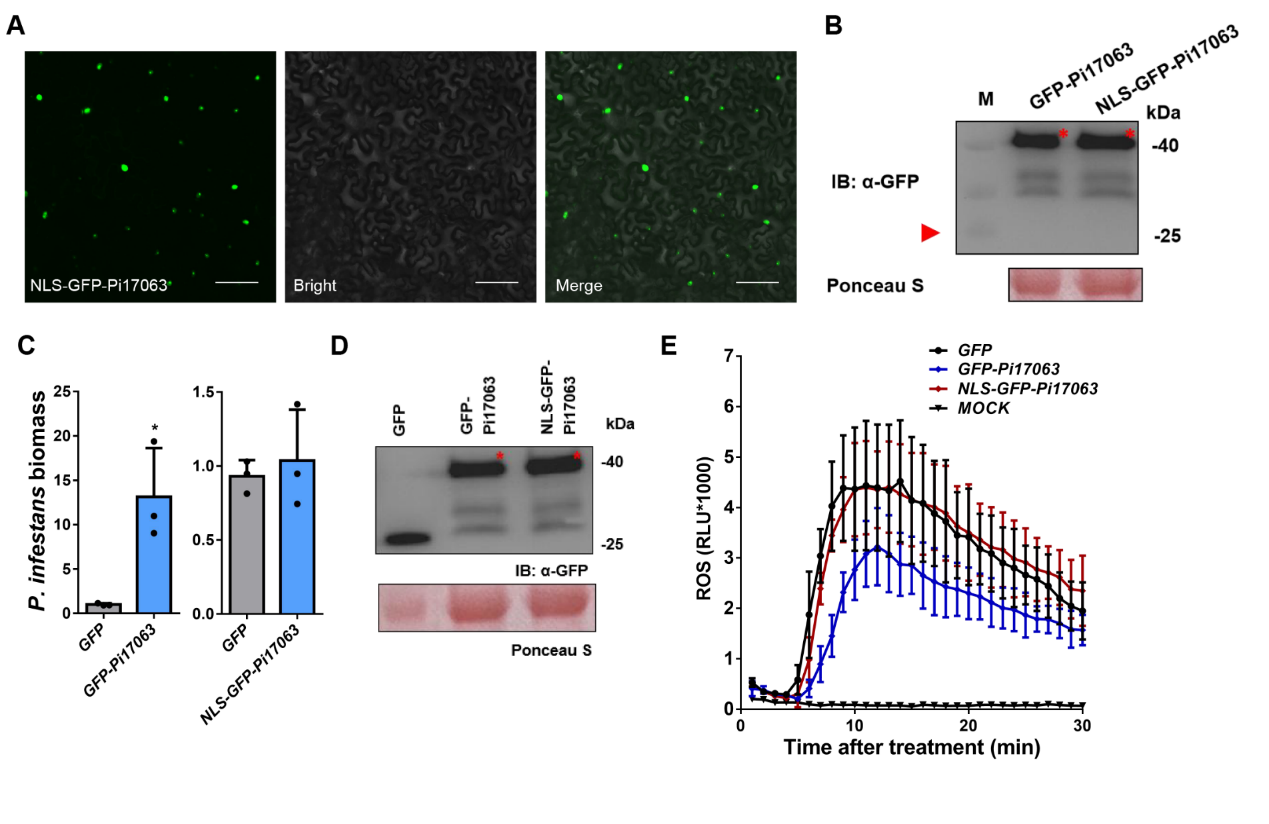


**Figure S2** **Nuclear-localized Pi17063 is abolished to inhibit plant PTI response.**

**(A)** NLS-GFP-Pi17063 re-localized to the nucleus. Scale bars = 20 μm. **(B, D)** protein expression and integrity of GFP, GFP-Pi17063 and NLS-GFP-Pi17063 were confirmed by Western blot. Ponceau S staining indicates amounts of protein samples loaded. Asterisks indicate bands of the expected sizes. The triangle indicates the size of free GFP. **(C)** *P. infestans* biomass in *N. benthamiana* leaves overexpressing *GFP*, *GFP-Pi17063* or *NLS-GFP-Pi17063* by genomic DNA qPCR at 7 dpi. Data are presented as the mean ± standard error (n=3). **P* < 0.05 (Student’s *t*-test). **(E)** luminol chemiluminescence assays to detect reactive oxygen species (ROS) bursts triggered by flg22 in leaves expressing *Pi17063, NLS-Pi17063*, or *GFP*. Data are shown as means ± SD (n=4). RLU, relative light units.


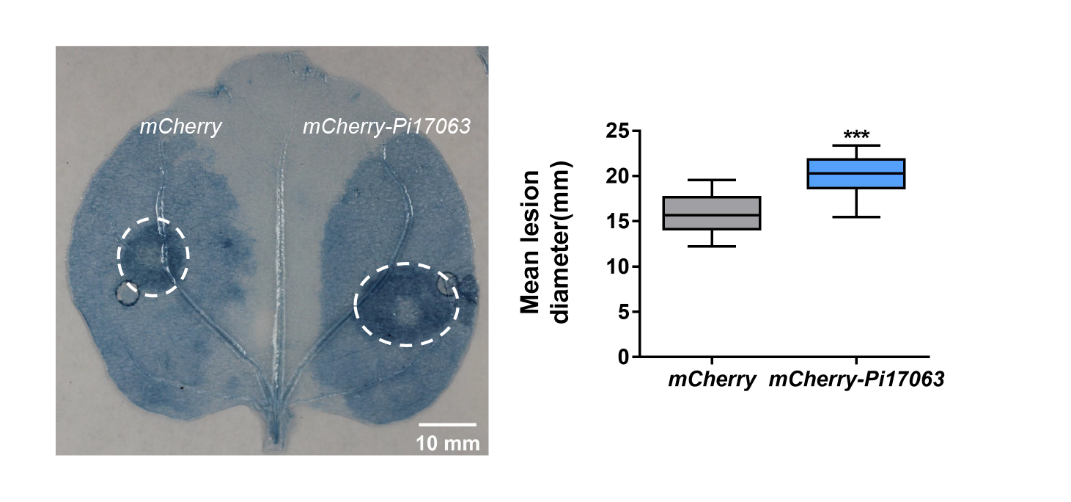


**Figure S3** **Overexpression of *mCherry-Pi17063* renders *N. benthamiana* more susceptible to *P. infestans*.**

Lesion images were taken (stained with trypan blue) and lesion diameters were measured at seven dpi. Dotted white circles indicate lesion areas. Lesion diameters were quantified and data are from 8 independent leaves. The upper quartile, median and lower quartile are shown in each box plot, while the bars outside the box indicate the maximum and minimum values. ****P* < 0.001 (Student’s *t*-test).


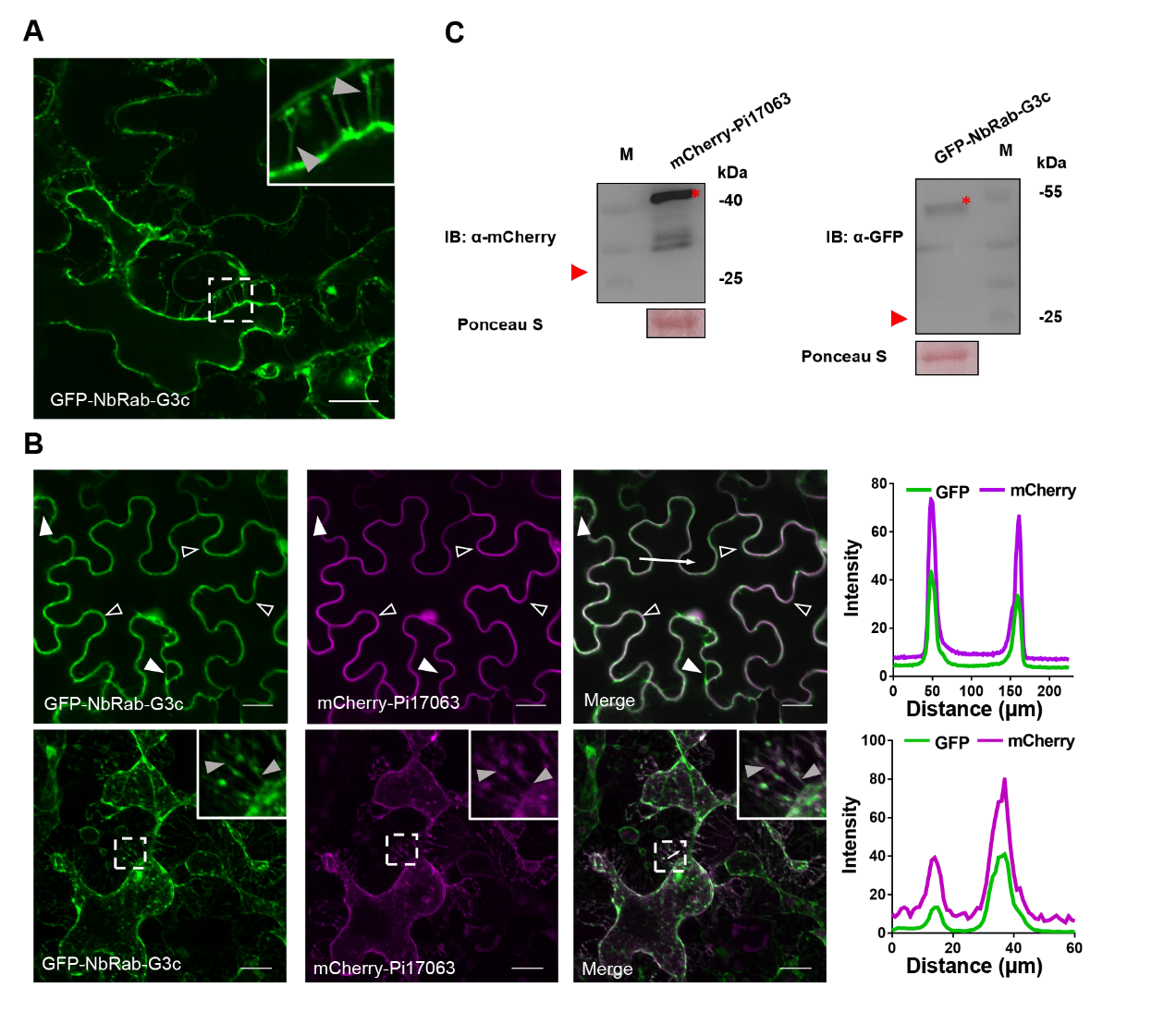


**Figure S4** **Pi17063 co-localizes with NbRab-G3c on PM.**

**(A)** hechtian strands of GFP-NbRab-G3c. **(B)** co-localization of GFP-NbRab-G3c and mCherry-Pi17063. **(C)** total protein was extracted from *N. benthamiana* after confocal observation, and the expression and integrity of GFP-NbRab-G3c and mCherry-Pi17063 were confirmed by Western blot. Ponceau S indicates protein samples loaded. Asterisks indicate bands of the expected sizes. The triangles indicate the sizes of free mCherry or free GFP. Confocal images information: Scale bars = 20 μm. The white dashed squares indicate local zoom-in insets. Fluorescence intensity was quantified along the transects (white line) with ImageJ. Examples of PM-, cytoplasmic- and hechtian strand-localizations are highlighted with open, white filled and gray filled triangles, respectively.


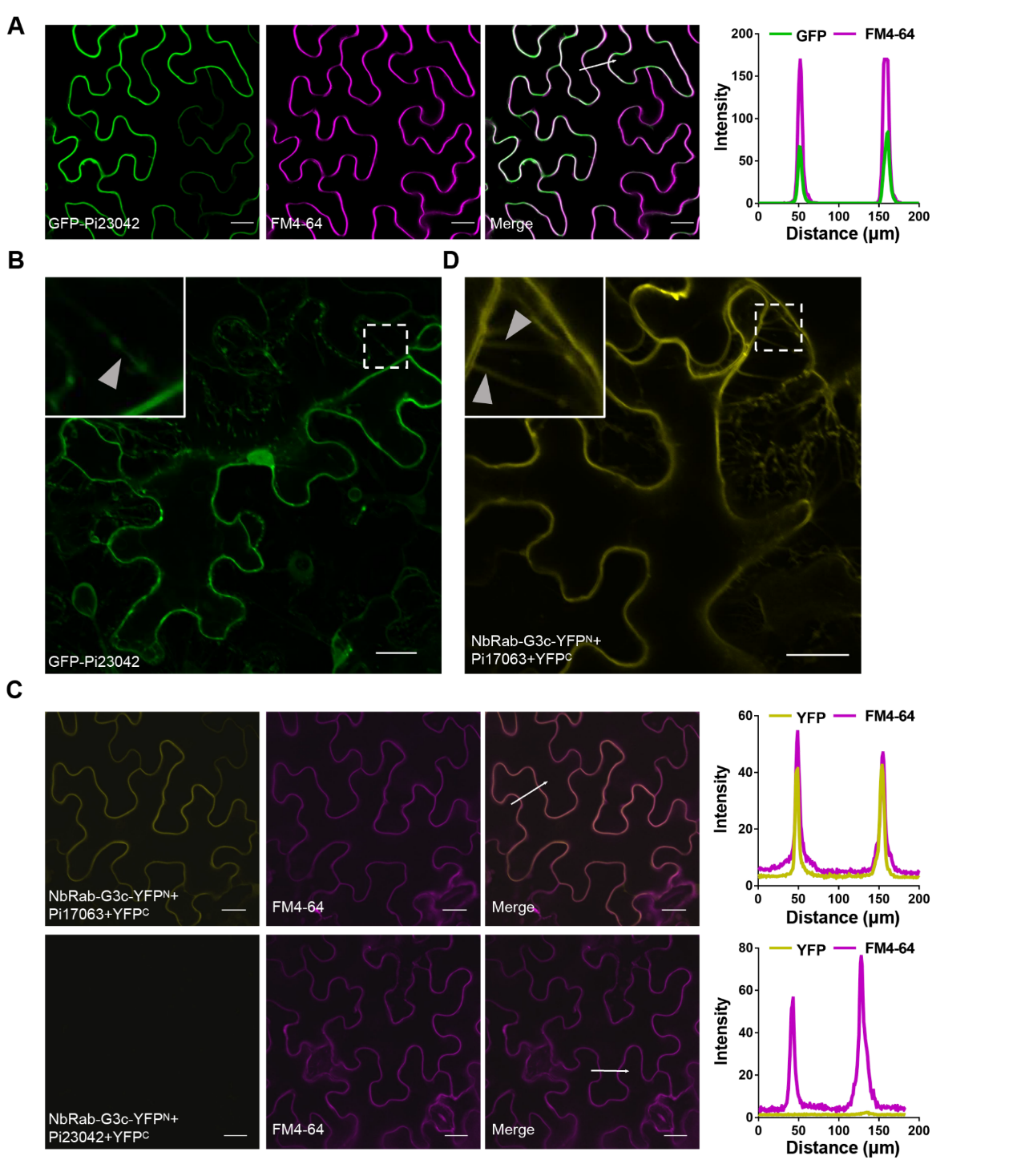


**Figure S5 Pi17063 interacts with NbRab-G3c on PM.**

**(A)** PM localization of Pi23042. **(B)** hechtian strands of GFP-Pi23042. **(C, D)** bimolecular fluorescence complementation (BiFC) assay showed Pi17063 interaction with NbRab-G3c on PM. Confocal images information: Scale bars = 20 μm. Fluorescence intensity was quantified along the transects (white line) with ImageJ. The white dashed squares indicate local zoom-in insets. Examples of hechtian strand-localization are highlighted with gray filled triangles.

**
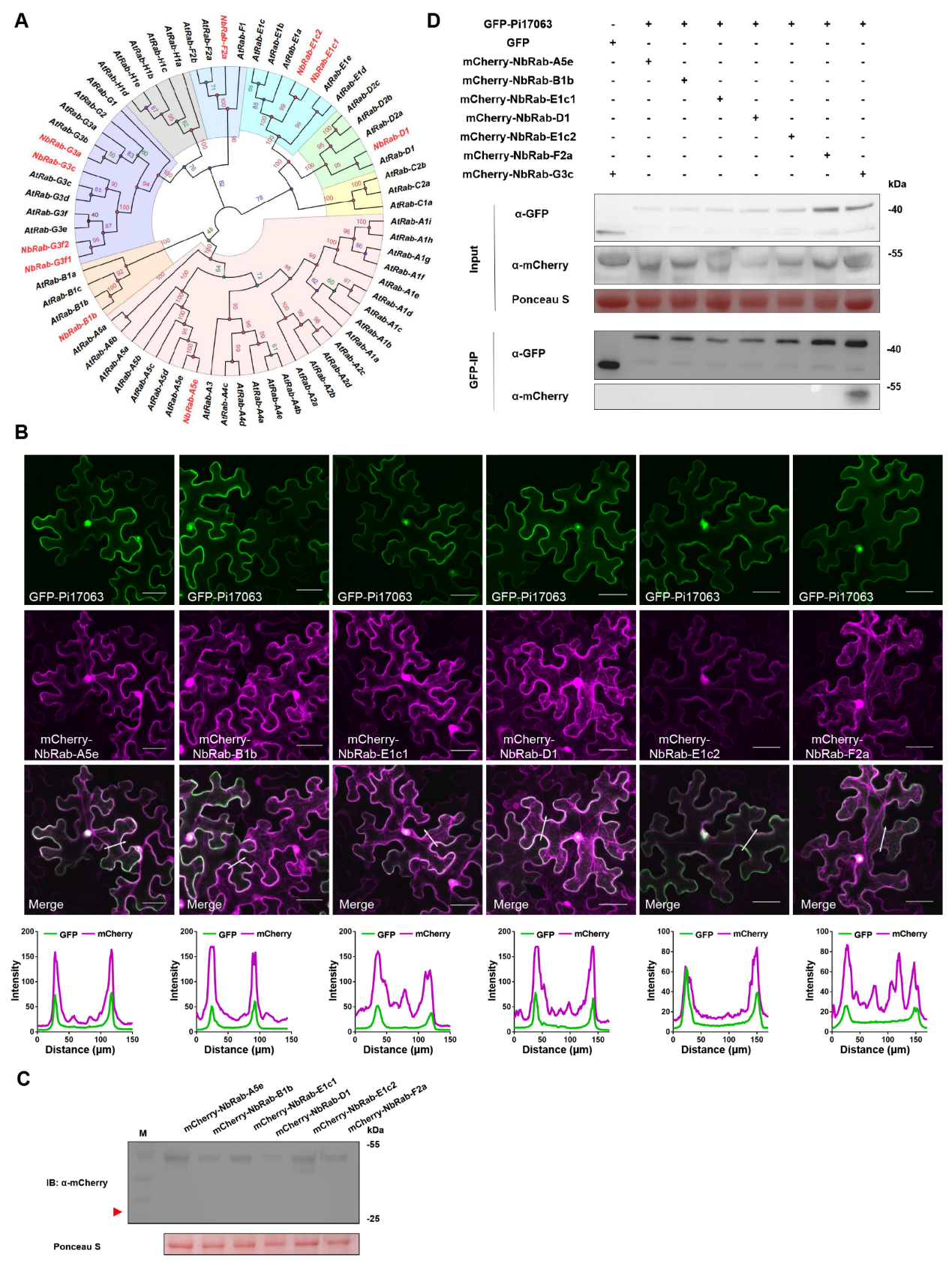
**

**Figure S6** **Pi17063 co-localizes with but cannot interact with NbRab-A - NbRab-F subfamily GTPases.**

**(A)** cladogram of Rab family members of *N. benthamiana* and *Arabidopsis thaliana*. The protein sequences of all *A. thaliana* Rab members were downloaded from TAIR (www.arabidopsis.org) and the gene family tree was Neighbor-Joining Tree which was constructed by MEGA. The branch labels are bootstrap value (the number of bootstrap replications is 1000), and tree branch lengths are transformed by cladogram. The names highlighted in red are NbRab members of *N. benthamiana* used in our study. **(B)** localization of NbRab subfamily proteins relative to Pi17063. Scale bars = 40 μm. Fluorescence intensity was quantified in the transect (white line) with ImageJ. **(C)** protein integrity test. Ponceau S indicates protein samples loaded. The position of free mCherry is marked with a triangle. **(D)** co-IP assays with GFP-trap beads to assess the interaction of Pi17063 with NbRab family proteins. Ponceau S indicates the loading of protein samples.


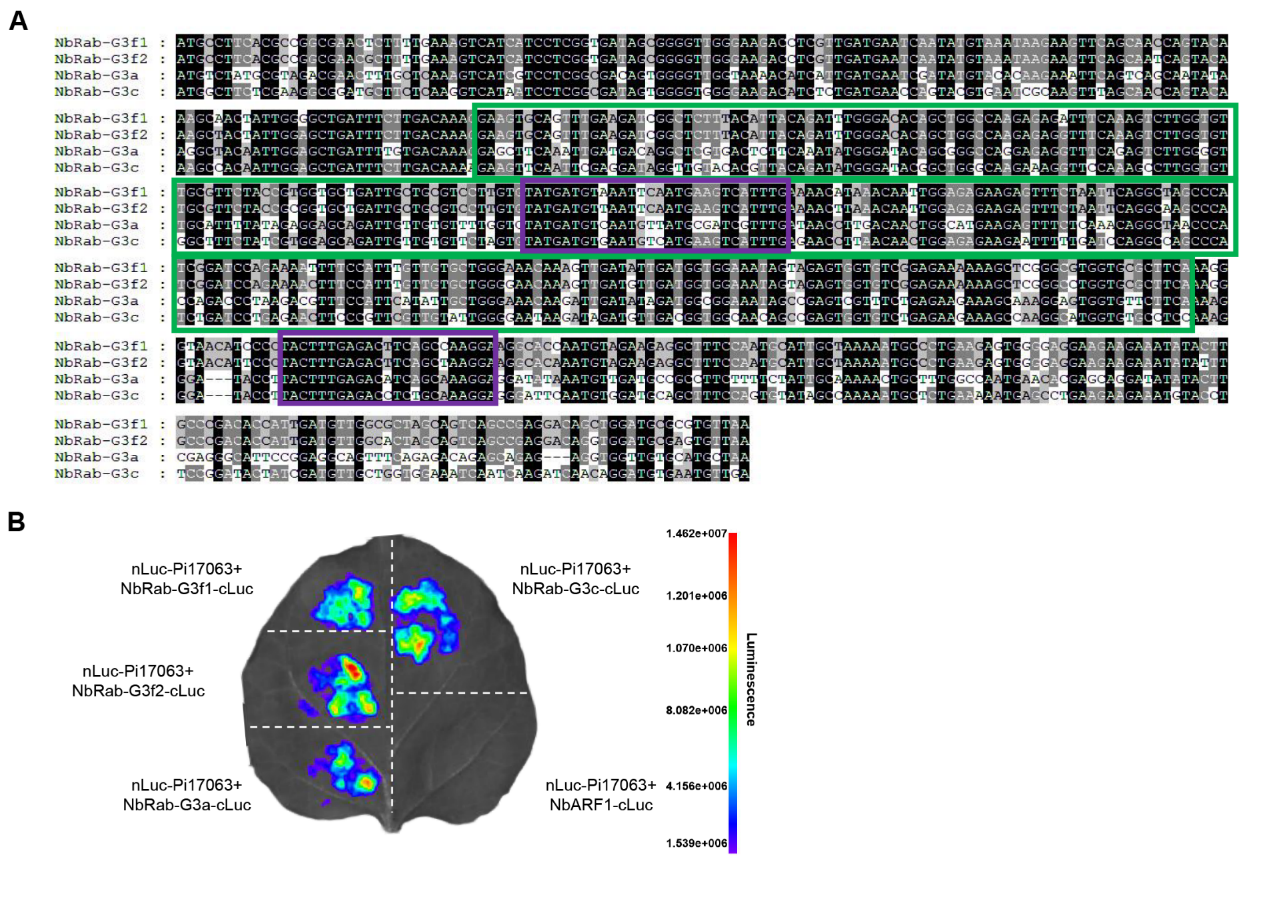


**Figure S7** **Pi17063 specifically interacts with the NbRab-G3 subfamily GTPases.**

**(A)** nucleotide sequences of the tested *Rab-G3* genes were aligned with MEGA7. Fully-conserved sites are shaded in black, and partially conserved sites are shaded in gray. The green box represents the fragment selected to trigger VIGS, and the purple boxes indicate the conserved regions used for primers for measurements of aggregate transcript levels of the *Rab-G3* genes. **(B)** luciferase complementation assay to assess the interaction between Pi17063 and NbRab-G3 subfamily proteins. Pi17063-nLuc + NbARF1-cLuc was set as negative control. Leaf images were taken at 3 dpi.


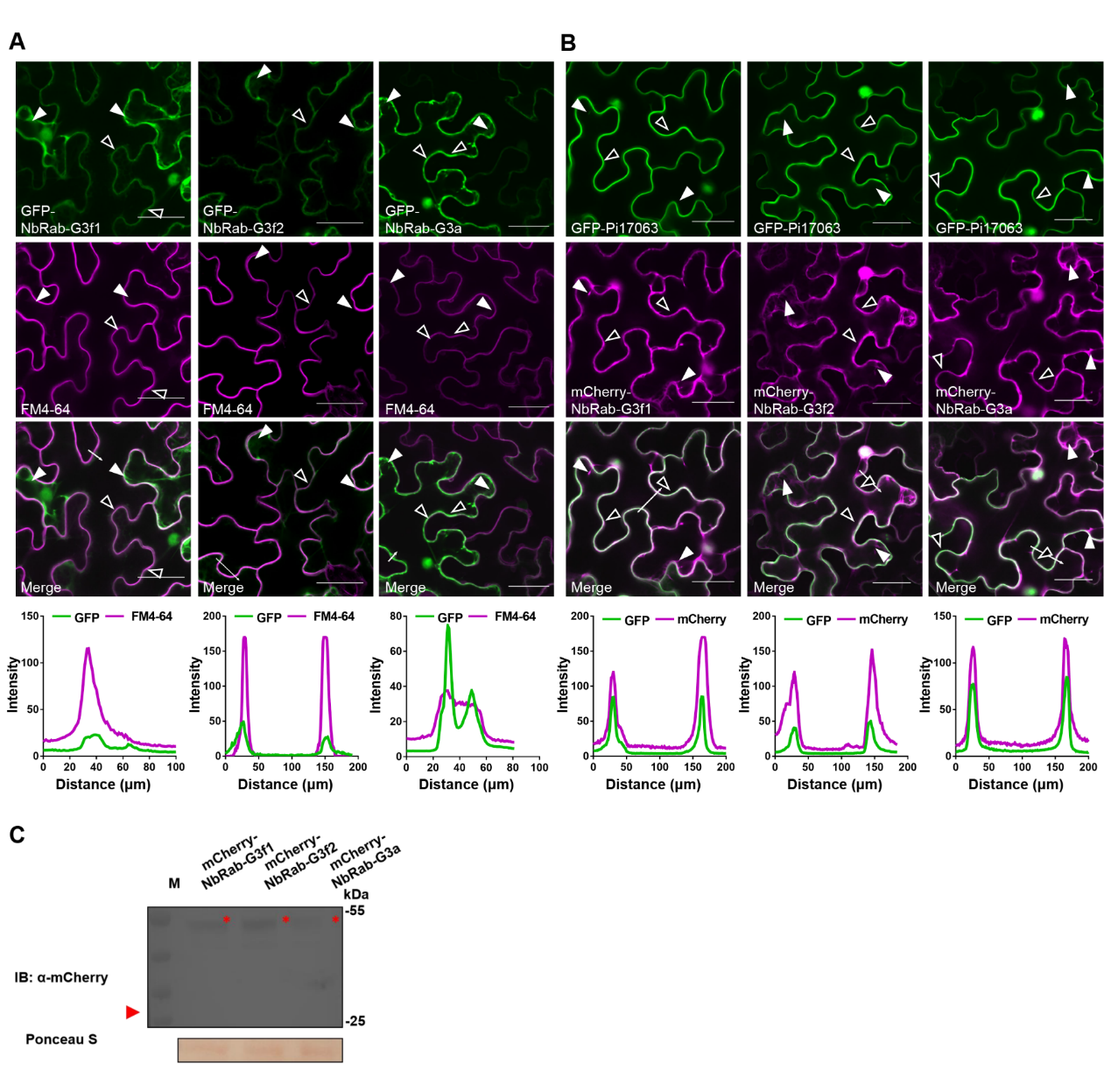


**Figure S8** **NbRab-G3 subfamily proteins localize and co-localize with Pi17063 on PM.**

**(A)** subcellular localization of NbRab-G3 proteins. **(B)** co-localization of GFP-Pi17063 with mCherry-NbRab-G3 proteins. **(C)** protein integrity of NbRab-G3 subfamily proteins. Ponceau S indicates protein samples loaded. The position of free mCherry is marked with a triangle. Confocal images information: Scale bars = 40 μm. Fluorescence intensity was analyzed along each transect (white line, right panels). The white dashed squares indicate local zoom-in insets. Examples of PM- and cytoplasmic- are highlighted with open and white filled triangles, respectively.


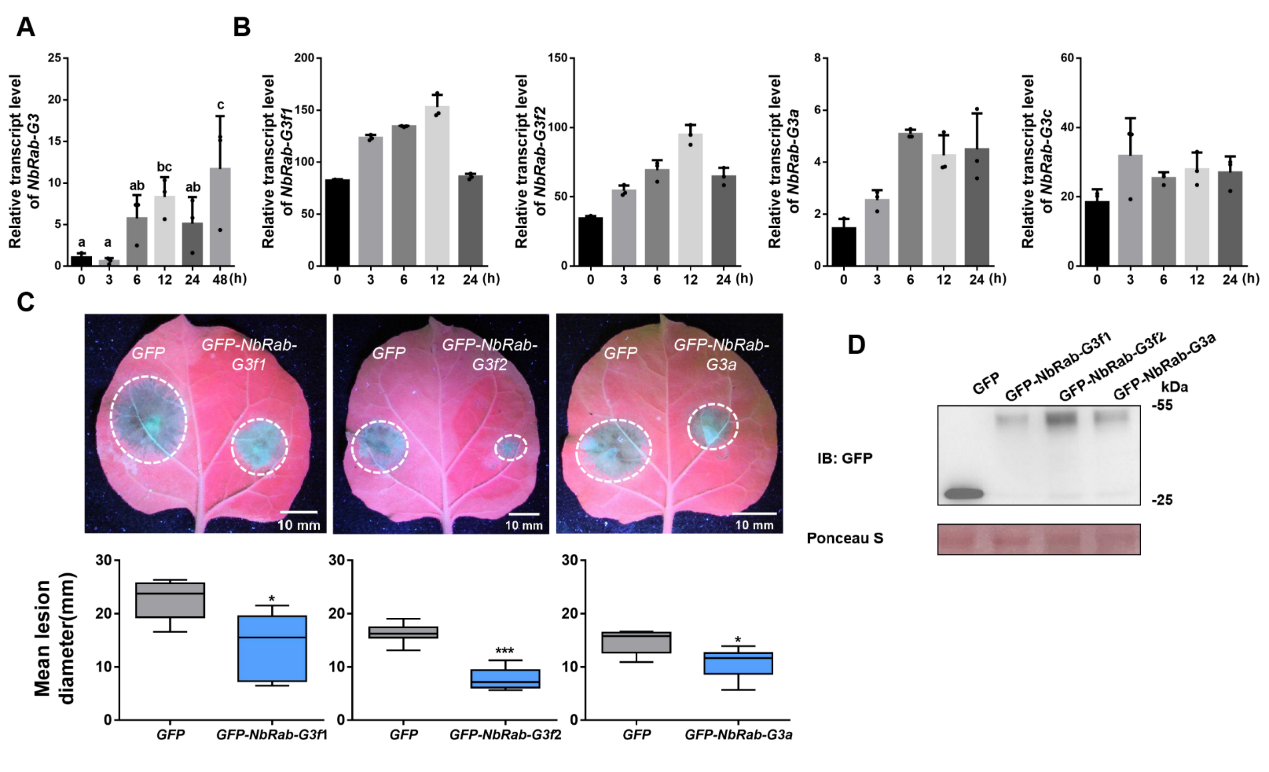


**Figure S9** **NbRab-G3 subfamily members NbRab-G3f1, NbRab-G3f2, and NbRab-G3a positively regulate plant immunity.**

**(A)** transcript levels of *NbRab-G3* genes. RNA was extracted from *N. benthamiana* leaves infected with *P. infestans* at multiple time points. Relative transcript levels of *NbRab-G3* genes were quantified by RT-qPCR with primers *q-Rab-G-F* and *q-Rab-G-R*, and with *NbActin* as an internal control. Data are shown as means ± SD (n=3) and were analyzed using Tukey’ s multiple comparisons test. Different letters indicate significant differences (*P* < 0.05). **(B)** transcript levels of different *NbRab-G3* genes obtained from transcriptome data. Data are presented as the mean ± standard error. **(C)** *N. benthamiana* leaves transiently over-expressing *NbRab-G3f1, NbRab-G3f2*, or *NbRab-G3a* were challenged with *P. infestans* zoospores at 1 dpi. *GFP* was used as a control. Infected leaves were photographed at seven dpi under UV light. Dotted white circles indicate lesion areas. Data are from at least 10 leaves. The upper quartile, median and lower quartile are shown in each box plot, while the bars outside the box indicate the maximum and minimum values. **P* < 0.05, ****P* < 0.001 (Student’s *t*-test). **(D)** protein expression and integrity were confirmed via Western blot. Ponceau S indicates amounts of protein samples loaded. The size of free GFP is evident in lane 1.


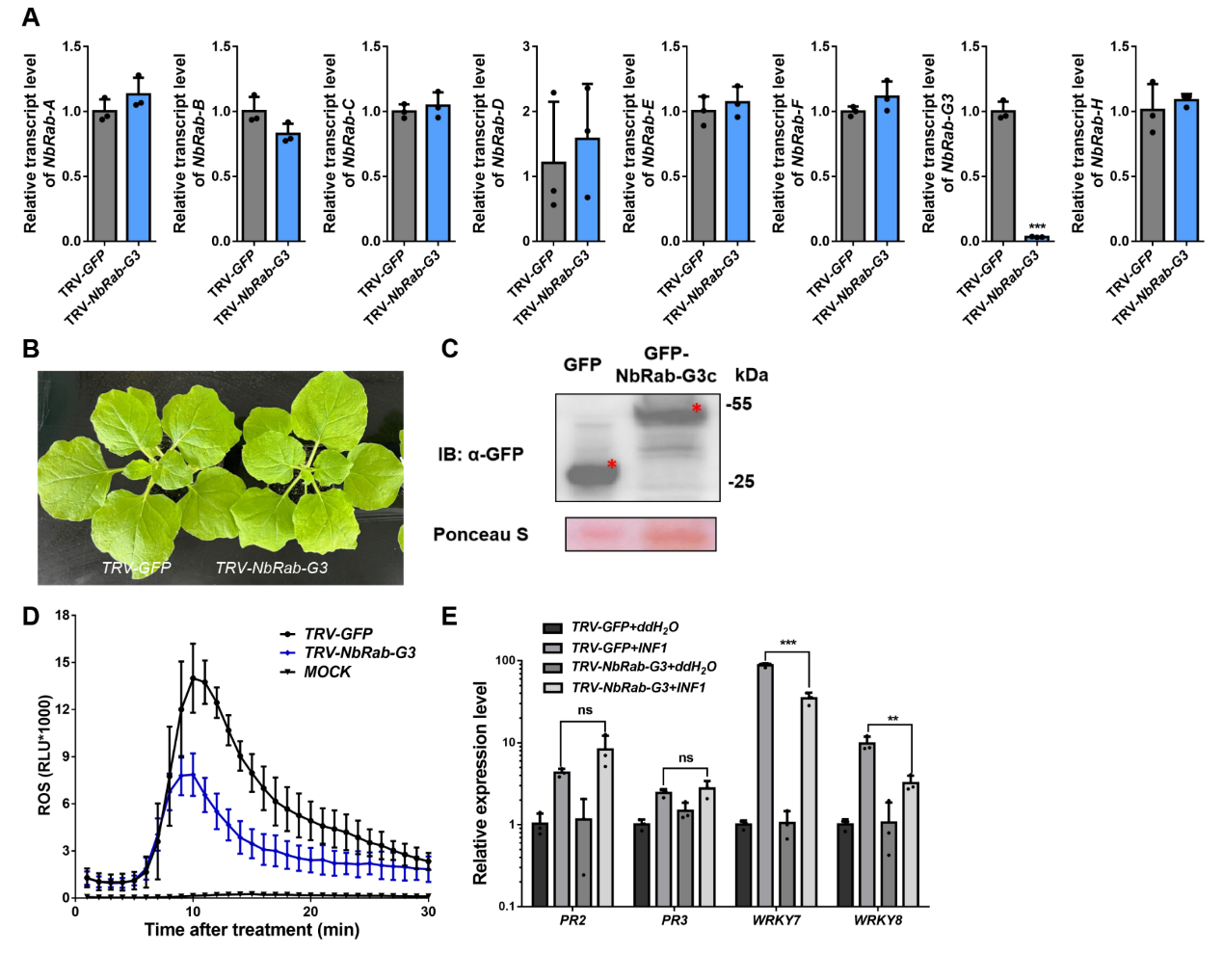


**Figure S10** ***NbRab-G3* genes positively regulate plant immunity and PTI responses.**

**(A)** silencing efficiency of *NbRab-G3* genes. Total RNA was extracted from six leaves with *NbRab-G3* silenced. Aggregate *NbRab-G3* transcript levels were measured with RT-qPCR and normalized to *NbActin*. The transcript levels of non-target genes (*NbRab-A, NbRab-B, NbRab-C, NbRab-D*, *NbRab-E, NbRab-F* and *NbRab-H*) were also tested. Data are presented as the mean ± standard error (n=3). ****P* < 0.001 (Student’s *t*-test). **(B)** growth phenotypes of *NbRab-G*-silenced plants were not affected. **(C)** protein expression and integrity were confirmed by Western blot. Ponceau S indicates protein samples loaded. Asterisks indicate bands of the expected sizes. The size of free GFP is evident in lane 1. **(D)** ROS burst levels in *TRV-GFP* and *TRV-NbRab-G3* leaves triggered with PAMP flg22. Data are shown as means ± SD (n=4). RLU, relative light units. Each test was repeated at least 3 times with consistent results. **(E)** attenuated PTI responses in *TRV-NbRab-G3* plants. Transcript levels of *PR2*, *PR3*, *WRKY7* and *WRKY8* were quantified via RT-qPCR, with *NbActin* as an internal control. Data are shown as means ± SD (n=3). ****P* < 0.001, ***P* < 0.01 (Student’s *t*-test).


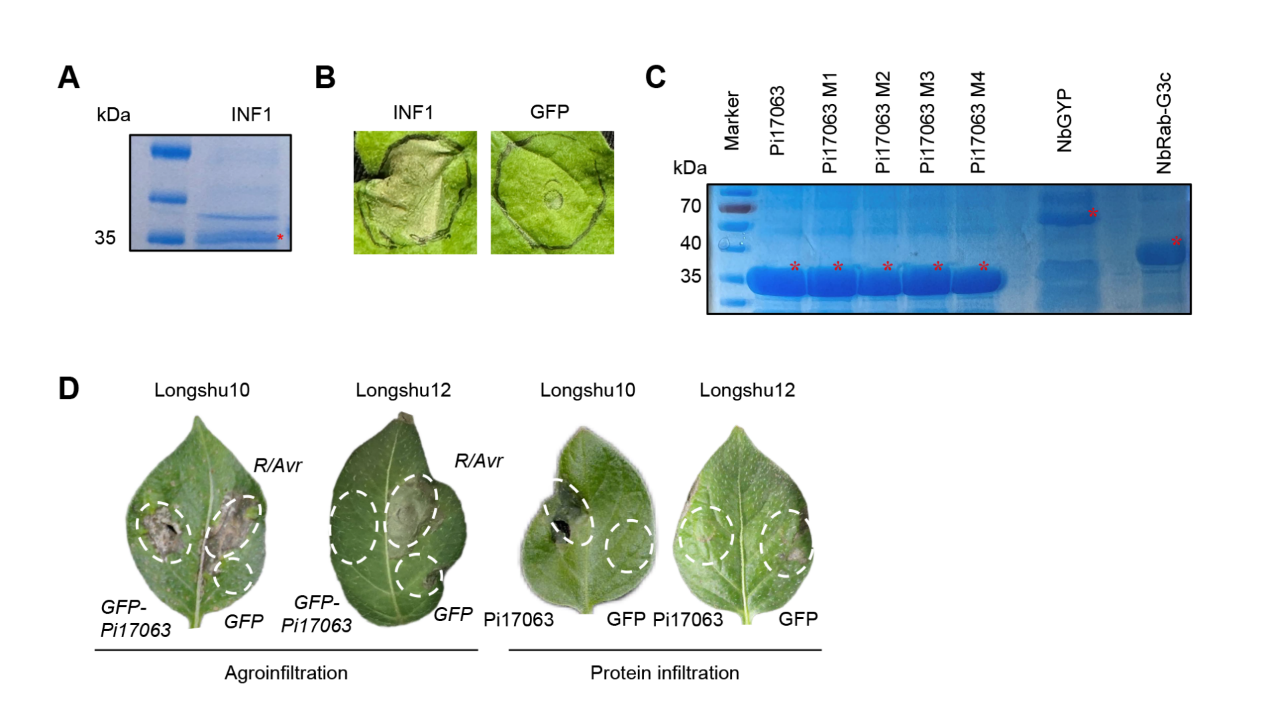


**Figure S11 Integrity and functional confirmation of recombinant proteins produced in *E. coli*.**

**(A, C)** integrity of recombinant proteins. Recombinant proteins produced in *E. coli*, were separated on 10% SDS-PAGE gels. The proteins were stained using Coomassie brilliant blue. Asterisks indicate bands of the expected sizes. **(B)** the *E. coli*-expressed recombinant protein INF1 was biologically functional. Recombinant INF1 and GFP proteins (1 µM) were directly infiltrated into *N. benthamiana* leaves. Images were taken at three dpi. **(D)** Pi17063 triggered a potato genotype-specific cell death. GFP was used as the negative control and the RB/Avrblb1 gene pair (R/Avr) as the positive control in the agroinfiltration assays. White dashed lines indicate areas of infiltration.


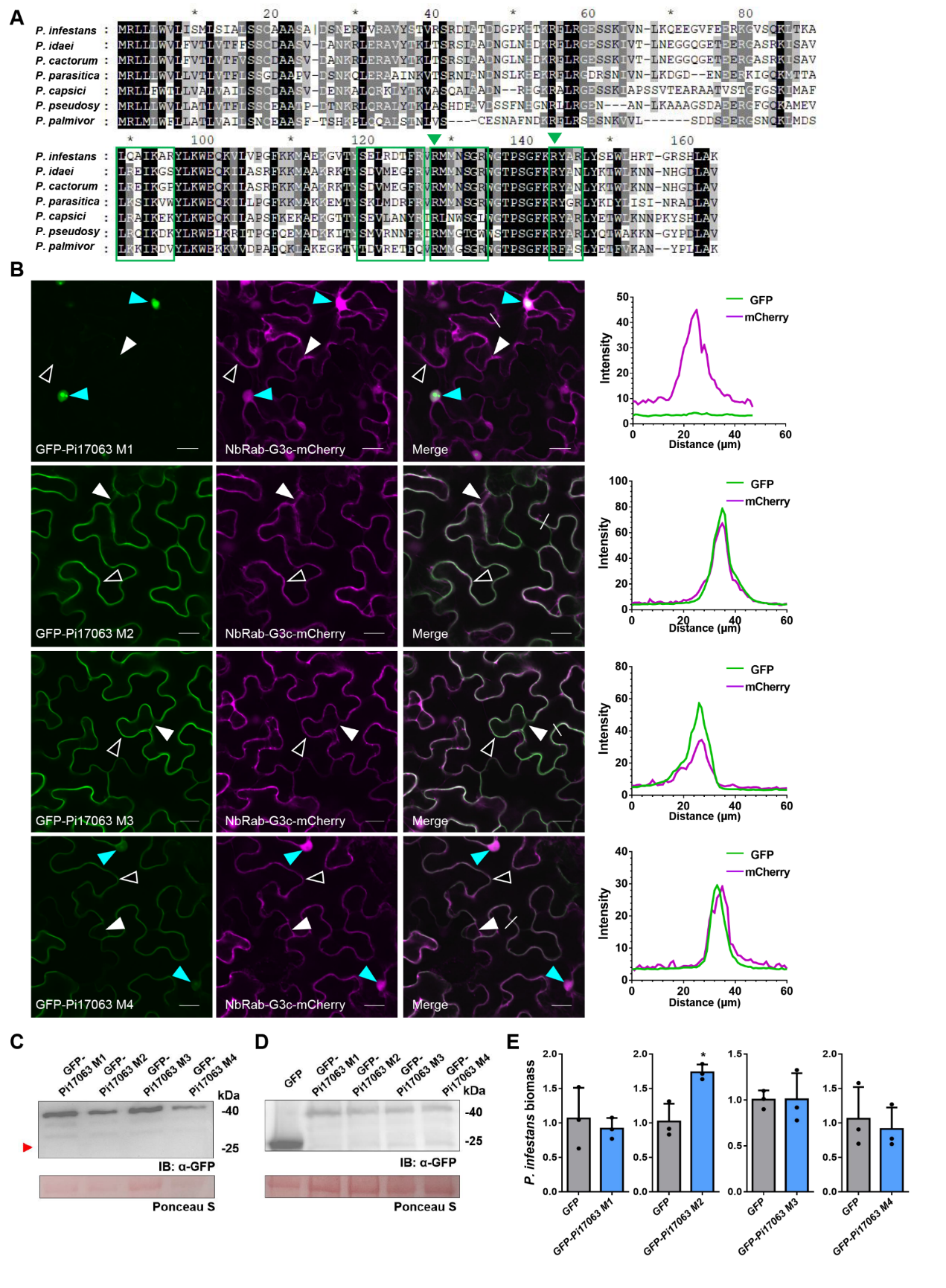


**Figure S12 Pi17063M2 retains host PM localization and promotes *P. infestans* colonization in *N. benthamiana*.**

**(A)** amino acid sequences of Pi17063 and its homologs in other *Phytophthora* species were aligned with MEGA7. The highly-conserved sites are shaded in black, and partially conserved residues are shaded in gray. The green boxes represent the sites that were mutated, M1 to M4 from left to right. Arrows indicate conserved arginine residues. **(B)** co-localization of GFP-Pi17063 mutant proteins with mCherry-NbRab-G3c. Scale bars = 20 μm. The fluorescence intensities at the transects (white line) were analyzed with ImageJ. Examples of PM-, cytoplasmic and nuclear localizations are highlighted by the open, white and cyan triangles, respectively. **(C, D)** total proteins were extracted from *N. benthamiana*, and protein expression and integrity were confirmed via Western blot. The size of free GFP is indicated by the triangle. **(E)** *P. infestans* biomass in *N. benthamiana* leaves overexpressing GFP-Pi17063 mutants was determined by genomic DNA qPCR at 7 dpi. Data are presented as the mean ± standard error (n=3). **P* < 0.05 (Student’s t-test).


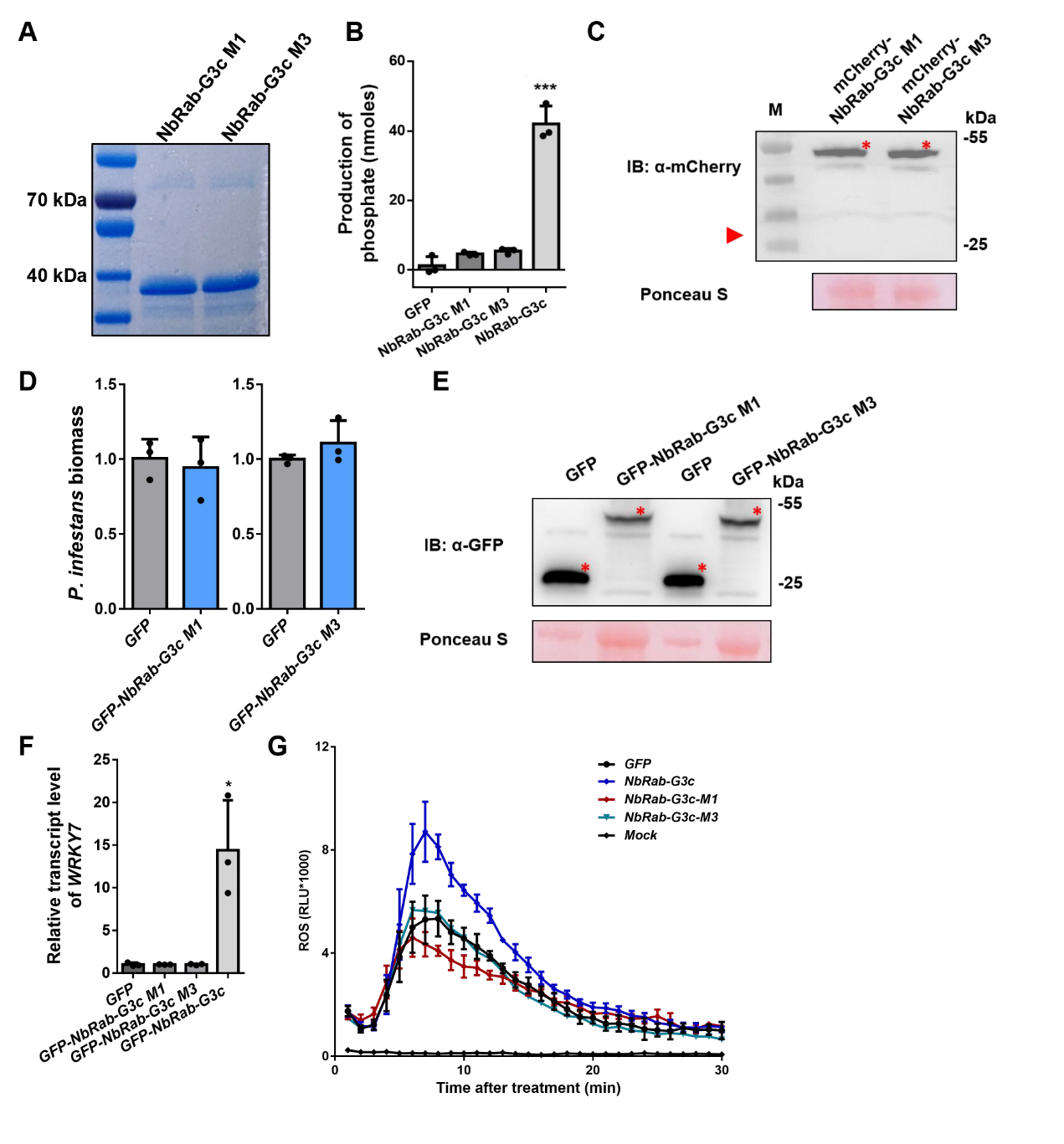


**Figure S13 NbRab-G3cM1 and NbRab-G3cM3 mainly localize on cytoplasm and PM, respectively, and both lose the ability to regulate plant immunity and PTI responses.**

**(A)** integrity of recombinant proteins. Recombinant proteins produced in *E. coli*, were separated on 10% SDS-PAGE gels and stained using Coomassie brilliant blue. Asterisks indicate bands of the expected sizes. **(B)** *in vitro* GTPase activities of NbRab-G3c mutants were quantified using a Plant GTP ELISA Kit. 40 μg of recombinant proteins (NbRab-G3c, NbRab-G3cM1, and NbRab-G3cM3) were incubated in the reaction solution for 30 minutes then the quantity of phosphate produced by the hydrolysis of GTP was measured. Data are presented as the mean ± standard error (n=3). **(C)** total protein was extracted from *N. benthamiana* and the expression and integrity of mCherry-NbRab-G3cM1 and mCherry-NbRab-G3cM3 were confirmed by Western blot. Red asterisks indicate the expected sizes of the expressed proteins. Triangles indicate the expected sizes of free mCherry. **(D)** *P. infestans* colonization levels were measured with qPCR targeting the genomic DNA of *PiUBC* and *NbActin*. Data are presented as the mean ± standard error. **(E)** protein expression and integrity of GFP, GFP-NbRab-G3cM1 and GFP-NbRab-G3cM3 were confirmed by Western blot. Ponceau S indicates the loading of protein samples. The expected sizes of the expressed proteins are indicated by red asterisks. **(F)** PTI responses of *N. benthamiana* leaves transiently expressed with *NbRab-G3c* mutant constructs. *N. benthamiana* leaves were treated with flg22 before total RNA was extracted and transcript levels of *WRKY7* were quantitated via RT-qPCR with *NbActin* as an internal control. Data are presented as the mean ± SD (n=3). **P* < 0.05 (Student’s *t*-test). **(G)** a luminol chemiluminescence assay used to detect ROS bursts. Data are shown as means ± SD (n=4). RLU, relative light units.


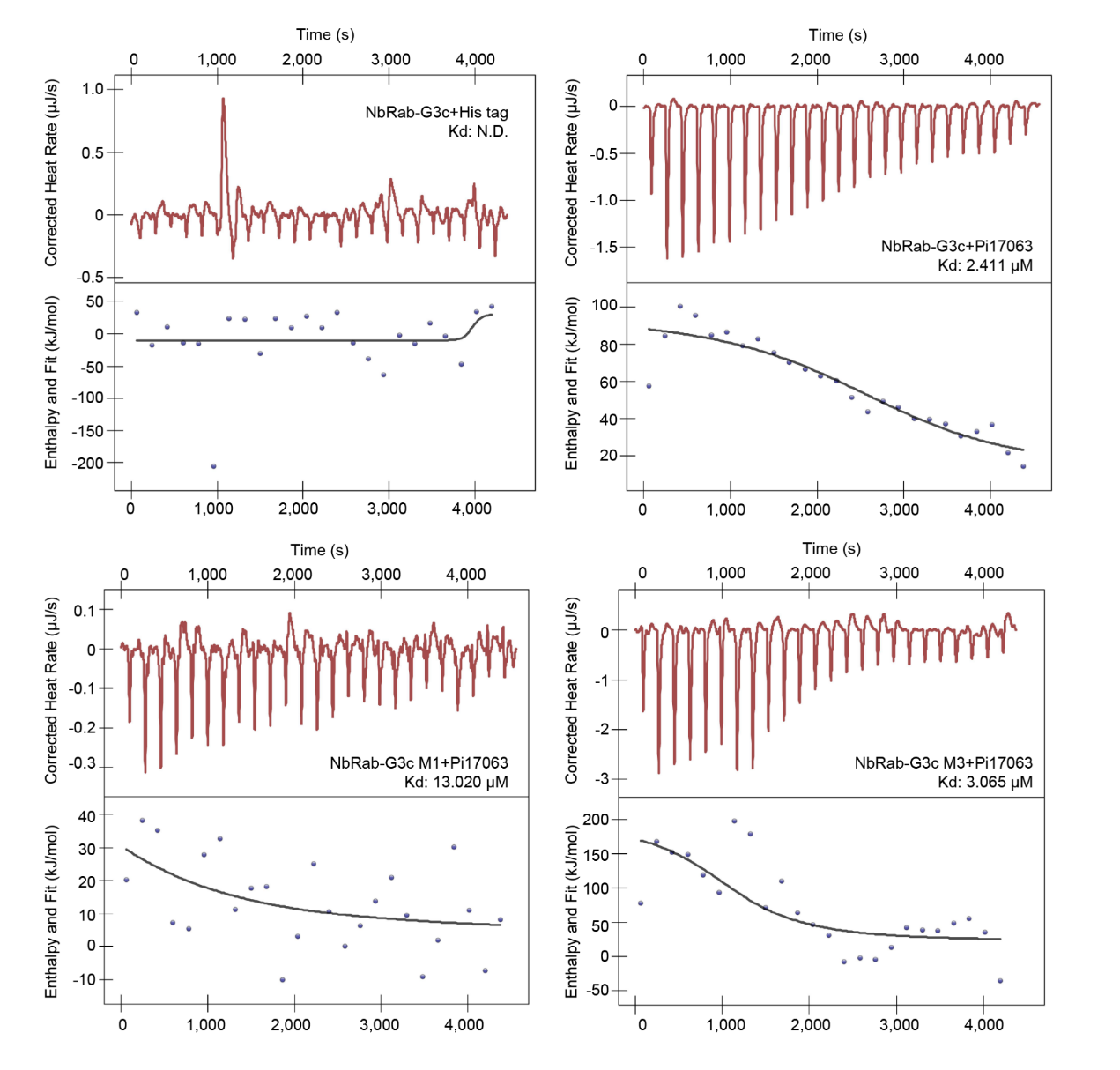


**Figure S14 Pi17063 preferentially interacts with the GTP-bound NbRab-G3c.**

Recombinant NbRab-G3c and its mutant proteins were titrated with identical concentrations of Pi17063 protein, respectively, in isothermal titration calorimetry assays. His tag was used as negative control. Kd, dissociation constant; N. D., Not Detectable.

**
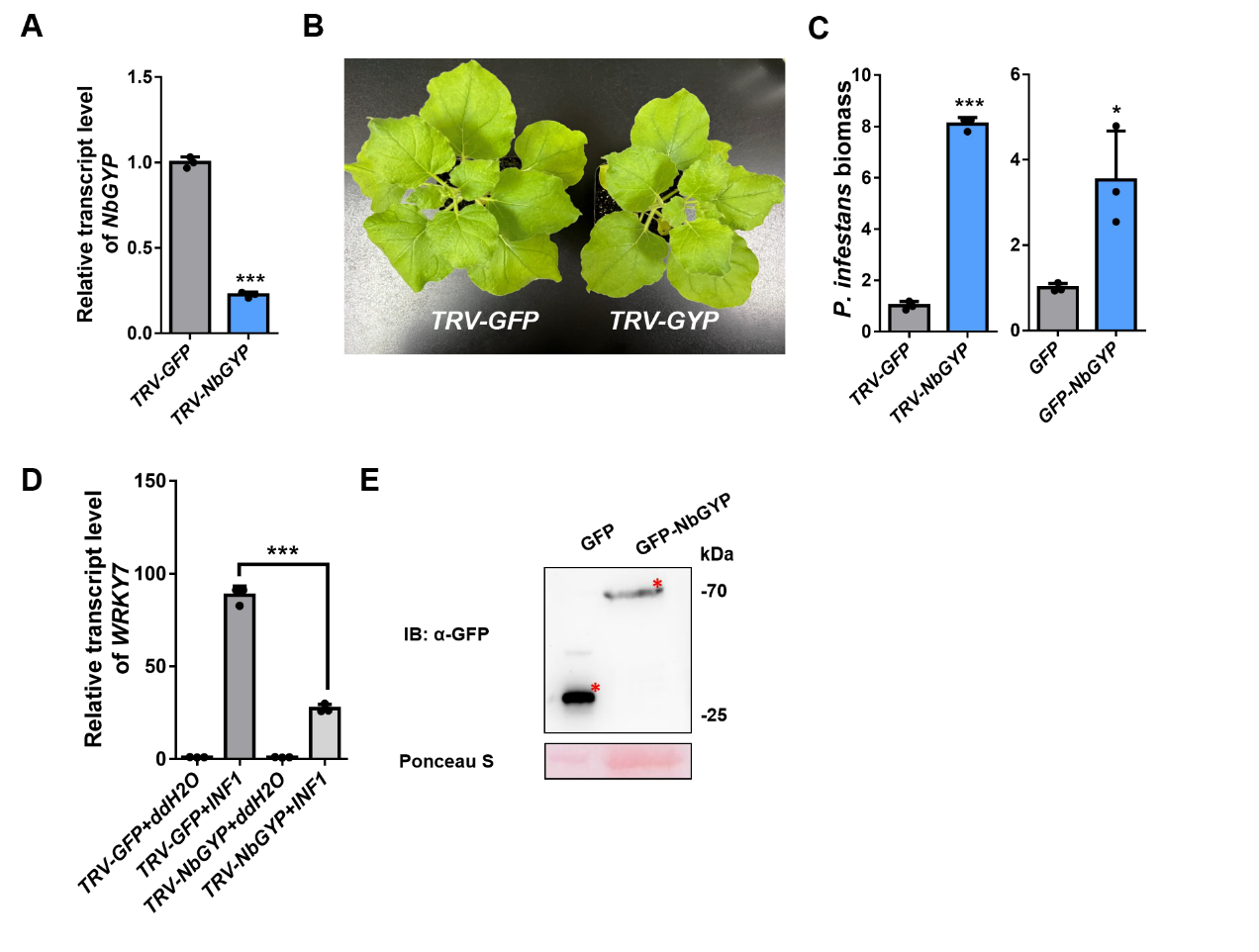
**

**Figure S15 Both silencing and overexpression of *NbGYP* render *N. benthamiana* more susceptible to *P. infestans*.**

**(A)** *NbGYP* silencing efficiency. Total RNA was extracted from five *NbGYP*-silenced leaves. Data are presented as the mean ± standard error (n=3). ****P* < 0.001 (Student’s *t*-test). **(B)** growth phenotypes of *NbGYP*-silenced plants were not affected. **(C)** *P. infestans* biomass in leaves silencing or overexpressing *NbGYP* at 7 dpi. Data are presented as the mean ± standard error (n=3). **P* < 0.05, ****P* < 0.001 (Student’s *t*-test). **(D)** transcript levels of *WRKY7* were quantified via RT-qPCR, with *NbActin* as an internal control. Data are presented as the mean ± standard error (n=3). ****P* < 0.001 (Student’s *t*-test). **(E)** total protein was extracted from *N. benthamiana* after confocal observation, and the protein expression and integrity were confirmed by Western blot. Ponceau S indicates amounts of protein samples loaded.


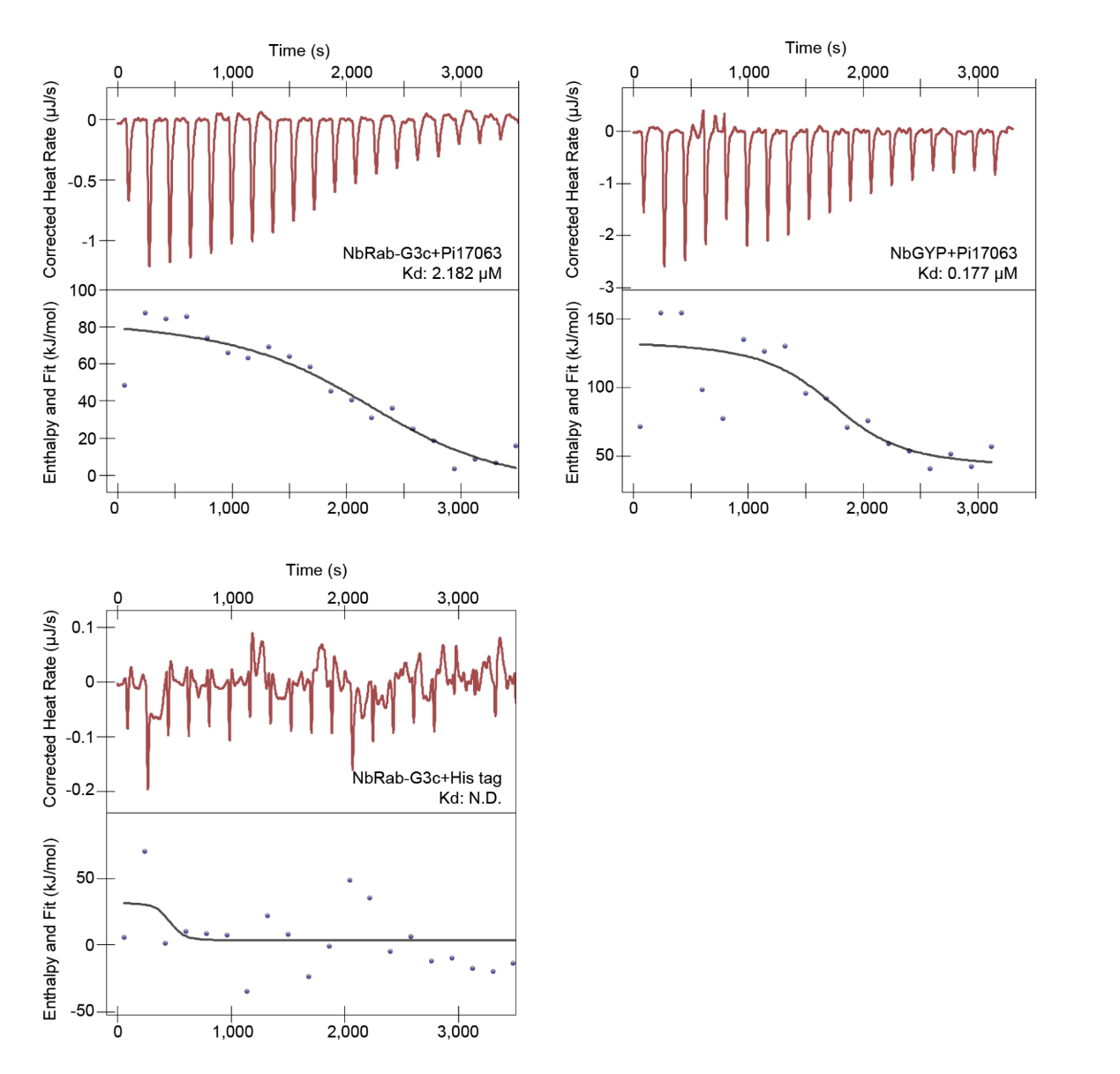


**Figure S16 Interaction intensity of Pi17063 with NbRab-G3c is slightly weaker than NbGYP.**

Recombinant NbRab-G3c protein was titrated with identical concentrations of Pi17063 or NbGYP proteins, respectively, in isothermal titration calorimetry assays. His tag was used as negative control. Kd, dissociation constant; N. D., Not Detectable.

**Table S1: Results of LC/MS-MS**

| Accession | Coverage | Peptides | Unique Peptides | MW [kDa] |
| --- | --- | --- | --- | --- |
| Niben101Scf02029g00004.1 sp | 42 | 10 | 4 | 35.2 |
| Niben101Scf12382g00021.1 sp | 40 | 5 | 1 | 28.3 |
| Niben101Scf01785g10010.1 sp | 32 | 5 | 1 | 105.1 |
| Niben101Scf03711g01021.1 sp | 31 | 2 | 2 | 10.3 |
| Niben101Scf02147g01007.1 sp | 26 | 4 | 1 | 25.4 |
| Niben101Scf02771g01007.1 sp | 21 | 4 | 1 | 57.7 |
| Niben101Scf08156g08017.1 sp | 21 | 1 | 1 | 11 |
| Niben101Scf06826g08015.1 sp | 17 | 2 | 1 | 37.5 |
| Niben101Scf02673g00001.1 sp | 16 | 1 | 1 | 14.5 |
| Niben101Scf00223g03023.1 sp | 15 | 1 | 1 | 15.8 |
| Niben101Scf36173g00001.1 sp | 14 | 1 | 1 | 14.6 |
| Niben101Scf00220g04015.1 sp | 13 | 1 | 1 | 20.5 |
| Niben101Scf02517g16014.1 sp | 12 | 1 | 1 | 26.4 |
| Niben101Scf00683g03020.1 sp | 12 | 1 | 1 | 19.8 |
| **Niben101Scf01374g03034.1** | **12** | **1** | **1** | **23** |
| Niben101Scf01433g08029.1 | 11 | 2 | 2 | 44.8 |
| Niben101Scf06919g00006.1 sp | 10 | 1 | 1 | 19.6 |
| Niben101Scf00063g10008.1 sp | 10 | 1 | 1 | 27.7 |
| Niben101Scf08020g01012.1 sp | 9 | 1 | 1 | 29.9 |
| Niben101Scf13411g05021.1 sp | 8 | 1 | 1 | 28.6 |
| Niben101Scf04053g03033.1 sp | 8 | 1 | 1 | 35.3 |
| Niben101Scf01276g00005.1 sp | 8 | 1 | 1 | 31.4 |
| Niben101Scf00444g01008.1 sp | 8 | 1 | 1 | 41.7 |
| Niben101Scf03580g01006.1 sp | 7 | 1 | 1 | 38.6 |
| Niben101Scf02159g01014.1 sp | 6 | 1 | 1 | 39.8 |
| Niben101Scf19423g00008.1 sp | 5 | 1 | 1 | 25.1 |
| Niben101Scf05950g00001.1 sp | 5 | 1 | 1 | 37.3 |
| Niben101Scf02112g00007.1 sp | 5 | 1 | 1 | 24.1 |
| Niben101Scf00167g04011.1 sp | 5 | 1 | 1 | 55.4 |
| Niben101Scf10400g00005.1 sp | 4 | 1 | 1 | 55.1 |
| Niben101Scf08898g00002.1 sp | 4 | 1 | 1 | 67.2 |
| Niben101Scf07123g00001.1 sp | 4 | 1 | 1 | 46.5 |
| Niben101Scf11178g01001.1 sp | 3 | 2 | 2 | 80.1 |
| Niben101Scf08590g00005.1 sp | 3 | 1 | 1 | 78.1 |
| Niben101Scf03321g01012.1 sp | 3 | 1 | 1 | 51.1 |
| Niben101Scf39332g00017.1 sp | 2 | 2 | 2 | 160.9 |
| Niben101Scf06228g00010.1 | 1 | 1 | 1 | 144.3 |

**Table S2: Primers used in this study**

**Note: The italic sequences are cleavage sites of restriction enzyme or homologous sequences to the respective vector.**

| Primer name | Primer sequence (5’-3’) | Experiments |
| --- | --- | --- |
| nluc-Pi17063-F | *acgagctcggtacccgggatcc*ATGGCTTCGGCTGATTCGAAT | Protein-protein interaction |
| nluc-Pi17063-R | *acgagctcggtacccgggatcc*CTTTGCAAGATGGGAGCGAC | Protein-protein interaction |
| NbRab-G3c-cluc-F | *ggggcggtacccgggatcc*ATGGCTTCTCGAAGGCGGAT | Protein-protein interaction |
| NbRab-G3c-cluc-R | *acgaaagctctgcaggtcgac*ACATTCACATCCTGTTGATCTTGAT | Protein-protein interaction |
| NbRab-G3f1-cluc-F | *ggggcggtacccgggatcc*ATGCCTTCACGCCGGCGA | Protein-protein interaction |
| NbRab-G3f1-cluc-R | *acgaaagctctgcaggtcgac*ACACGCGCATCCAGCTGTC | Protein-protein interaction |
| NbRab-G3f2-cluc-F | *ggggcggtacccgggatcc*ATGCCTTCACGCCGGCGAAC | Protein-protein interaction |
| NbRab-G3f2-cluc-R | *acgaaagctctgcaggtcgac*ACACTCGCATCCACCTGTC | Protein-protein interaction |
| NbRab-G3a-cluc-F | *ggggcggtacccgggatcc*ATGTCTATGCGTAGACGAACTTTG | Protein-protein interaction |
| NbRab-G3a-cluc-R | *acgaaagctctgcaggtcgac*GCATGCACAACCACCTCTCT | Protein-protein interaction |
| YFPN-NbRab-G3c-F | *gggcccaggcctactagt*ATGGCTTCTCGAAGGCGGAT | Protein-protein interaction |
| YFPN-NbRab-G3c-R | *gggagcggtaccctcgag*ACATTCACATCCTGTTGATCTTGAT | Protein-protein interaction |
| YFPC-Pi17063-F | gggcccaggcctactagtATGGCTTCGGCTGATTCGAAT | Protein-protein interaction |
| YFPC-Pi17063-R | gggagcggtaccctcgagCTTTGCAAGATGGGAGCGAC | Protein-protein interaction |
| YFPC-Pi23042-F | *gggcccaggcctactagt*ATGGCACACATCGAGCTCCA | Protein-protein interaction |
| YFPC-Pi23042-R | *gggagcggtaccctcgag*TGACCTGAACCATGAGTCATTCT | Protein-protein interaction |
| TRV2-NbRab-G3-F | *cggaattc*GAAGTTCAATTCGAGGATAGGTT | VIGS |
| TRV2-NbRab-G3-R | *gctctaga*GGAGGCACACCATGCCTTG | VIGS |
| TRV2-NbGYP-F | *cggaattc*TCCTTCGGAGATTACGAGGAA | VIGS |
| TRV2-NbGYP-R | *gctctaga*GGATTCAGTTTGGCAAAAACAATGA | VIGS |
| PET-Pi17063-F/PET-Pi17063M-F | *tgttccaggggcccgccatgg*CTATGGCTTCGGCTGATTCGAAT | Recombinant protein preparation |
| PET-Pi17063-R/PET-Pi17063M-R | *gtggtggtggtggtgctcgag*TTACTTTGCAAGATGGGAGCGA | Recombinant protein preparation |
| PET-NbRab-G3c-F/PET-NbRab-G3cM-F | *tgttccaggggcccgccatgg*CTATGGCTTCTCGAAGGCGGAT | Recombinant protein preparation |
| PET-NbRab-G3c-R/PET-NbRab-G3cM-F | *gtggtggtggtggtgctcgag*ACATTCACATCCTGTTGATCTTGAT | Recombinant protein preparation |
| PET-NbGYP-F | *tgttccaggggcccgccatgg*CTATGGGAGAAGTCAGGAGGTTA | Recombinant protein preparation |
| PET-NbGYP-R | *gtggtggtggtggtgctcgag*TTAGCCAGCTGATTTAACACGTA | Recombinant protein preparation |
| NbRab-A5e-mCherry-F | *tttggagaggacacgctcgag*ATGGATTCATCAGATAATGAAAGT | Overexpression analysis |
| NbRab-A5e-mCherry-R | *atccttataatccatgaattc*CCTTGAACAACAAGAATAGCCTT | Overexpression analysis |
| NbRab-B1b-mCherry-F | *tttggagaggacacgctcgag*ATGTCGTACGCCTATCTCTTCAA | Overexpression analysis |
| NbRab-B1b-mCherry-R | *atccttataatccatgaattc*ACTGCAACAACCCCCTCCTT | Overexpression analysis |
| NbRab-E1c1-mCherry-F | *tttggagaggacacgctcgag*ATGGAAGCATCATCATCATCGC | Overexpression analysis |
| NbRab-E1c1-mCherry-R | *atccttataatccatgaattc*TGGATTTGTTGATGCATCAGTTTC | Overexpression analysis |
| NbRab-D1-mCherry-F | *tttggagaggacacgctcgag*ATGAGCAACGAATACGATTACTTGT | Overexpression analysis |
| NbRab-D1-mCherry-R | *atccttataatccatgaattc*GCCACAACAGTTGCTCTTCTC | Overexpression analysis |
| NbRab-E1c2-mCherry-F | *tttggagaggacacgctcgag*ATGGAGCCTGGAAAGTCCAAA | Overexpression analysis |
| NbRab-E1c2-mCherry-R | *atccttataatccatgaattc*AGAGCCACAGCAAGCTGATTTT | Overexpression analysis |
| NbRab-F2a-mCherry-F | *tttggagaggacacgctcgag*ATGGCAACCGGTGGAAACAAG | Overexpression analysis |
| NbRab-F2a-mCherry-R | *atccttataatccatgaattc*AGAGCAACAAGAAGCACTAGCT | Overexpression analysis |
| NbGYP-mCherry-F | *tttggagaggacacgctcgag*ATGGGAGAAGTCAGGAGGTTA | Overexpression analysis |
| NbGYP-mCherry-R | *atccttataatccatgaattc*GCCAGCTGATTTAACACGTAATTTA | Overexpression analysis |
| GFP-NLS-Pi17063-F1 | ATGCCGAAAAAAAAACGTAAAGTGATGTCGAAGGGCGAGGAG | Mutants preparation |
| GFP-NLS-Pi17063-F2 | *gacgagctgtacaaggaattcATGCCGAAAAAAAAACGTAAAGTGAT* | Mutants preparation |
| GFP-Pi17063-F/GFP-Pi17063M-F/mCherry-Pi17063-F | *gacgagctgtacaaggaattcATGTCGAAGGGCGAGGAG* | Overexpression analysis |
| GFP-Pi17063-R/GFP-NLS-Pi17063-R/GFP-Pi17063M-R/mCherry-Pi17063-R | *cattaaagcaggactctagacttaacTTACTTTGCAAGATGGGAGCGA* | Overexpression analysis |
| GFP-NbRab-G3c-F/GFP-NbRab-G3c M-F/mCherry-NbRab-G3c-F/mCherry-NbRab-G3c M-F | *gacgagctgtacaaggaattcATGGCTTCTCGAAGGCGGAT* | Overexpression analysis |
| GFP-NbRab-G3c-R/GFP-NbRab-G3c M-R/mCherry-NbRab-G3c-R/mCherry-NbRab-G3c M-R | *cattaaagcaggactctagacttaacACATTCACATCCTGTTGATCTTGAT* | Overexpression analysis |
| mCherry/GFP-NbRab-G3f1-F | *gacgagctgtacaaggaattcATGCCTTCACGCCGGCGA* | Overexpression analysis |
| mCherry/GFP-NbRab-G3f1-R | *cattaaagcaggactctagacttaacACACGCGCATCCAGCTGTC* | Overexpression analysis |
| mCherry/GFP-NbRab-G3f2-F | *gacgagctgtacaaggaattcATGCCTTCACGCCGGCGAAC* | Overexpression analysis |
| mCherry/GFP-NbRab-G3f2-R | *cattaaagcaggactctagacttaacACACTCGCATCCACCTGTC* | Overexpression analysis |
| mCherry/GFP-NbRab-G3a-F | *gacgagctgtacaaggaattcATGTCTATGCGTAGACGAACTTTG* | Overexpression analysis |
| mCherry/GFP-NbRab-G3a-R | *cattaaagcaggactctagacttaacGCATGCACAACCACCTCTCT* | Overexpression analysis |
| mCherry/GFP-NbGYP-F | *gacgagctgtacaaggaattcATGGGAGAAGTCAGGAGGTTA* | Overexpression analysis |
| mCherry/GFP-NbGYP-R | *cattaaagcaggactctagacttaacGCCAGCTGATTTAACACGTAATTTA* | Overexpression analysis |
| NbRab-G3cM-F | ATGGCTTCTCGAAGGCGGAT | Overexpression analysis |
| NbRab-G3cM1-R1 | TGGTTCATCAGAGAGTTCTTCC | Mutants preparation |
| NbRab-G3cM1-F2 | GGGAAGAACTCTCTGATGAAC | Mutants preparation |
| NbRab-G3cM-R | ACATTCACATCCTGTTGATCTTG | Overexpression analysis |
| NbRab-G3cM3-R1 | AACCTTTCGAGCCCAGCCG | Mutants preparation |
| NbRab-G3cM3-F2 | GGGCTCGAAAGGTTCCAAAG | Mutants preparation |
| Pi17063M-F1 | ATGTCGAAGGGCGAGGAG | Overexpression analysis |
| Pi17063M1-F2 | GCAGCAGCAGCAGCAGCAGCATACCTCAAATGGGAGCAAAAAGTA | Overexpression analysis |
| Pi17063M1-R1 | TGCTGCTGCTGCTGCTGCTGCTGCCTTAGTGAGCTTTTGGCT | Overexpression analysis |
| Pi17063M2-F2 | GCAGCAGCAGCAGCAGCAGCAGCAGTTAGAATGATGAACAGTGGCC | Overexpression analysis |
| Pi17063M2-R1 | TGCTGCTGCTGCTGCTGCTGCTGCGTACGTTACCCCCTTTTCGG | Overexpression analysis |
| Pi17063M3-F2 | GCAGCAGCAGCAGCAGCAGCATGGGGCACACCGTCGGG | Overexpression analysis |
| Pi17063M3-R1 | TGCTGCTGCTGCTGCTGCTGCAACACGAAAGGTGTCTCTGAGT | Overexpression analysis |
| Pi17063M4-F2 | GCAGCAGCAGCACTCTACAGTGAGTGGCTGCA | Overexpression analysis |
| Pi17063M4-R1 | TGCTGCTGCTGCCTTGAACCCCGACGGTGTG | Overexpression analysis |
| Pi17063M-R2 | TTACTTTGCAAGATGGGAGCGA | Overexpression analysis |
| q-NbActin-F | TCCATGCTCAATGGGATACT | qPCR |
| q-NbActin-R | TTCAACCCCTTGTCTGTGAT | qPCR |
| q-PiUBC-F | CATCAATCGGCGTATCTGICTCA | qPCR |
| q-PiUBC-R | CACCAAGTCGGCGAATAGCAC | qPCR |
| q-NbWRKY7-F | CAGCCAAACGCGTTTCTTGA | qPCR |
| q-NbWRKY7-R | GAACGACAGGCATGCTGTTG | qPCR |
| q-NbWRKY8-F | AACAATGGTGCCAATAATGC | qPCR |
| q-NbWRKY8-R | TGCATATCCTGAGAAACCATT | qPCR |
| q-NbPR2-F | AGGTGTTTGCTATGGAATGC | qPCR |
| q-NbPR2-R | TCTGTACCCACCATCTTGC | qPCR |
| q-NbPR3-F | CAATGCCTTTATCAATGCTG | qPCR |
| q-NbPR3-R | AGTAGTCACCTGGGCTACCT | qPCR |
| q-Rab-A-F | CCTCGATGCAACAAACGTAAACA | qPCR |
| q-Rab-A-R | GATCCATCGGTACCATTATCGA | qPCR |
| q-Rab-B-F | GGAGGCCTCTGCTAAAACAG | qPCR |
| q-Rab-B-R | CCTTGAGGAGCAGCTCCAT | qPCR |
| q-Rab-C-F | CACAATTGTTTATTTCTTGAATGTAGTG | qPCR |
| q-Rab-C-R | CATTTTTCGCTATGTCGAGACTT | qPCR |
| q-Rab-D-F | AAGACTCCATTAACGTGGAGCA | qPCR |
| q-Rab-D-R | TGGAGTCTTTTGCACTCGTCT | qPCR |
| q-Rab-E-F | ACTAGCCGACGAATATGGCATTA | qPCR |
| q-Rab-E-R | CTTTGAGCAGCTCCTGCTG | qPCR |
| q-Rab-F-F | AAACATATGCCCAGGAGAATGG | qPCR |
| q-Rab-F-R | AACAAGAAGCACTAGCTGGTGTT | qPCR |
| q-Rab-G-F | TGATGTGAATGTCATGAAGTCATTTG | qPCR |
| q-Rab-G-R | TCCTTTGCAGAGGTCTCAAAGTA | qPCR |
| q-Rab-H-F | AGATGCTAAGGCTCGTGAATTTG | qPCR |
| q-Rab-H-R | CTTCAAATTAACGTCCACCATATCT | qPCR |
| q-NbGYP-F | AGAGGTAGAGGACAAGGGC | qPCR |
| q-NbGYP-R | ATATCTTATACCCGGATTCAGTTTG | qPCR |

**Table S3: Statistical analysis tables**

**Statistical analysis table of lesion diameter**

| **Figure** | **Sample** | **Difference between means** | **95% confidence interval** | ***P* Value** | **Significance** | **Test** |
| --- | --- | --- | --- | --- | --- | --- |
| Figure 3C | TRV-NbRab-G3 to TRV-GFP | 5.500 ± 1.291 | 2.788 to 8.212 | 0.0005 | *** | t test/two-tailed |
| Figure 6D | TRV-NbGYP to TRV-GFP | 5.838 ± 1.565 | 2.593 to 9.084 | 0.0012 | ** | t test/two-tailed |
|  |  |  |  |  |  |  |
| **Figure** | **Sample** | **Mean of differences** | **95% confidence interval** | ***P* Value** | **Significance** | **Test** |
| Figure 1D | GFP-Pi17063 to GFP | 4.833 | 3.002 to 6.665 | < 0.0001 | *** | paired t test/two-tailed |
| Figure 1D | NLS-GFP-Pi17063 to GFP | 0.8095 | -0.9246 to 2.544 | 0.3316 | ns | paired t test/two-tailed |
| Figure 3C | GFP-NbRab-G3c to GFP | -5.333 | -6.944 to -3.723 | < 0.0001 | *** | paired t test/two-tailed |
| Figure 4G | GFP-Pi7063 M1 to GFP | -1.561 | -3.244 to 0.1219 | 0.0653 | ns | paired t test/two-tailed |
| Figure 4G | GFP-Pi7063 M2 to GFP | 7.557 | 4.093 to 11.02 | 0.0008 | *** | paired t test/two-tailed |
| Figure 4G | GFP-Pi7063 M3 to GFP | 0.3419 | -3.087 to 3.770 | 0.8266 | ns | paired t test/two-tailed |
| Figure 4G | GFP-Pi7063 M4 to GFP | 0.3204 | -2.587 to 3.228 | 0.8088 | ns | paired t test/two-tailed |
| Figure 5F | GFP-NbRab-G3c M1 to GFP | -0.05556 | -2.338 to 2.227 | 0.9525 | ns | paired t test/two-tailed |
| Figure 5F | GFP-NbRab-G3c M3 to GFP | 0.05556 | -1.878 to 1.990 | 0.944 | ns | paired t test/two-tailed |
| Figure 6D | GFP-NbGYP to GFP | 2.762 | 1.012 to 4.512 | 0.0084 | ** | paired t test/two-tailed |
| Figure S3 | mCherry-Pi17063 to mCherry | 4.384 | 2.678 to 6.089 | 0.0005 | *** | paired t test/two-tailed |
| Figure S9C | GFP-NbRab-G3f1 to GFP | -8.446 | -15.40 to -1.489 | 0.0262 | * | paired t test/two-tailed |
| Figure S9C | GFP-NbRab-G3f2 to GFP | -8.59 | -10.91 to -6.273 | < 0.0001 | *** | paired t test/two-tailed |
| Figure S9C | GFP-NbRab-G3a to GFP | -4.002 | -6.628 to -1.377 | 0.0112 | * | paired t test/two-tailed |

**Statistical analysis table of *Phytophthora* biomass**

| **Figure** | **Sample** | **Difference between means** | **95% confidence interval** | ***P* Value** | **Significance** | **Test** |
| --- | --- | --- | --- | --- | --- | --- |
| Figure 3D | TRV-NbRab-G3 to TRV-GFP | 3.895 ± 0.1662 | 3.433 to 4.356 | < 0.0001 | *** | t test/two-tailed |
| Figure 3D | GFP-NbRab-G3c to GFP | -0.7927 ± 0.03383 | -0.8866 to -0.6988 | < 0.0001 | *** | t test/two-tailed |
| Figure S2C | GFP-Pi17063 to GFP | 12.13 ± 3.174 | 3.315 to 20.94 | 0.0188 | * | t test/two-tailed |
| Figure S2C | NLS-GFP-Pi17063 to GFP | 0.1056 ± 0.2091 | -0.4750 to 0.6862 | 0.6401 | ns | t test/two-tailed |
| Figure S12E | GFP-Pi7063 M1 to GFP | -0.1465 ± 0.2719 | -0.9015 to 0.6086 | 0.6188 | ns | t test/two-tailed |
| Figure S12E | GFP-Pi7063 M2 to GFP | 0.7148 ± 0.1622 | 0.2644 to 1.165 | 0.0116 | * | t test/two-tailed |
| Figure S12E | GFP-Pi7063 M3 to GFP | 0.003633 ± 0.1743 | -0.4803 to 0.4876 | 0.9844 | ns | t test/two-tailed |
| Figure S12E | GFP-Pi7063 M4 to GFP | -0.1497 ± 0.3209 | -1.041 to 0.7414 | 0.6652 | ns | t test/two-tailed |
| Figure S13D | GFP-NbRab-G3c M1 to GFP | -0.06137 ± 0.1391 | -0.4476 to 0.3249 | 0.6819 | ns | t test/two-tailed |
| Figure S13D | GFP-NbRab-G3c M3 to GFP | 0.1081 ± 0.08773 | -0.1355 to 0.3516 | 0.2855 | ns | t test/two-tailed |
| Figure S15C | TRV-NbGYP to TRV-GFP | 7.087 ± 0.1764 | 6.597 to 7.576 | < 0.0001 | *** | t test/two-tailed |
| Figure S15C | GFP-NbGYP to GFP | 2.526 ± 0.6644 | 0.6816 to 4.371 | 0.0191 | * | t test/two-tailed |

**Statistical analysis table of GTPase content**

| **Figure** | **Sample** | **Difference between means** | **95% confidence interval** | ***P* Value** | **Significance** | **Test** |
| --- | --- | --- | --- | --- | --- | --- |
| Figure S13B | NbRab-G3c M1 to GFP | 3.364 ± 1.528 | -0.8784 to 7.606 | 0.0925 | ns | t test/two-tailed |
| Figure S13B | NbRab-G3c M3 to GFP | 4.171 ± 1.569 | -0.1845 to 8.526 | 0.0565 | ns | t test/two-tailed |
| Figure S13B | NbRab-G3c to GFP | 40.77 ± 3.325 | 31.54 to 50.01 | 0.0003 | *** | t test/two-tailed |

**Statistical analysis table of silencing efficiency**

| **Figure** | **Sample** | **Difference between means** | **95% confidence interval** | ***P* Value** | **Significance** | **Test** | **Note** |
| --- | --- | --- | --- | --- | --- | --- | --- |
| Figure S10A | TRV-NbRab-G3 to TRV-GFP | 0.1297 ± 0.09035 | -0.1211 to 0.3805 | 0.2245 | ns | t test/two-tailed | Primer:qRab-A |
| Figure S10A | TRV-NbRab-G3 to TRV-GFP | -0.1760 ± 0.07715 | -0.3903 to 0.03816 | 0.0846 | ns | t test/two-tailed | Primer:qRab-B |
| Figure S10A | TRV-NbRab-G3 to TRV-GFP | 0.04487 ± 0.06630 | -0.1392 to 0.2289 | 0.5357 | ns | t test/two-tailed | Primer:qRab-C |
| Figure S10A | TRV-NbRab-G3 to TRV-GFP | 0.3690 ± 0.7318 | -1.663 to 2.401 | 0.6406 | ns | t test/two-tailed | Primer:qRab-D |
| Figure S10A | TRV-NbRab-G3 to TRV-GFP | 0.06686 ± 0.09472 | -0.1961 to 0.3298 | 0.5192 | ns | t test/two-tailed | Primer:qRab-E |
| Figure S10A | TRV-NbRab-G3 to TRV-GFP | 0.1138 ± 0.07105 | -0.08343 to 0.3111 | 0.1844 | ns | t test/two-tailed | Primer:qRab-F |
| Figure S10A | TRV-NbRab-G3 to TRV-GFP | -0.9685 ± 0.04311 | -1.088 to -0.8488 | < 0.0001 | *** | t test/two-tailed | Primer:qRab-G |
| Figure S10A | TRV-NbRab-G3 to TRV-GFP | 0.07494 ± 0.1184 | -0.2538 to 0.4037 | 0.5611 | ns | t test/two-tailed | Primer:qRab-H |
| Figure S15A | TRV-NbGYP to TRV-GFP | -0.7748 ± 0.02082 | -0.8326 to -0.7170 | < 0.0001 | *** | t test/two-tailed |  |

**Statistical analysis table of** **PTI-related Gene expression**

| **Figure** | **Sample** | **Difference between means** | **95% confidence interval** | ***P* Value** | **Significance** | **Test** | **Note** |
| --- | --- | --- | --- | --- | --- | --- | --- |
| Figure S10E | TRV-NbRab-G3c to TRV-GFP | 3.966 ± 2.279 | -2.361 to 10.29 | 0.1568 | ns | t test/two-tailed | PR2(INF1 treatment) |
| Figure S10E | TRV-NbRab-G3c to TRV-GFP | 0.3355 ± 0.4103 | -0.8036 to 1.475 | 0.4594 | ns | t test/two-tailed | PR3(INF1 treatment) |
| Figure S10E | TRV-NbRab-G3c to TRV-GFP | -53.43 ± 4.493 | -65.91 to -40.96 | 0.0003 | *** | t test/two-tailed | WRKY7(INF1 treatment) |
| Figure S10E | TRV-NbRab-G3c to TRV-GFP | -6.572 ± 1.277 | -10.12 to -3.027 | 0.0068 | ** | t test/two-tailed | WRKY8(INF1 treatment) |
| Figure S13F | GFP-NbRab-G3c M1 to GFP | 0.0005242 ± 0.1208 | -0.3347 to 0.3358 | 0.9967 | ns | t test/two-tailed | WRKY7(Flg22 treatment) |
| Figure S13F | GFP-NbRab-G3c M3 to GFP | -0.002092 ± 0.1288 | -0.002092 ± 0.1288 | 0.9878 | ns | t test/two-tailed |  |
| Figure S13F | GFP-NbRab-G3c to GFP | 13.39 ± 3.378 | 4.008 to 22.77 | 0.0166 | * | t test/two-tailed |  |
| Figure S15D | TRV-NbGYP to TRV-GFP | -60.99 ± 3.094 | -69.58 to -52.40 | < 0.0001 | *** | t test/two-tailed | WRKY7(INF1 treatment) |

**Statistical analysis table of** **GTPase activity**

| **Figure** | **Sample** | ***P* Value** | **Significance** | **Test** |
| --- | --- | --- | --- | --- |
| Figure 4A | NbRab-G3c+GFP to NbRab-G3c+Pi17063(0.05μM) | 0.4254 | ns | Tukey’s honestly significant difference (HSD) test |
| Figure 4A | NbRab-G3c+GFP to NbRab-G3c+Pi17063(0.1μM) | 0.1595 | ns | Tukey’s honestly significant difference (HSD) test |
| Figure 4A | NbRab-G3c+GFP to NbRab-G3c+Pi17063(0.2μM) | 0.0238 | * | Tukey’s honestly significant difference (HSD) test |
| Figure 4A | NbRab-G3c+GFP to Pi17063 | 0.0181 | * | Tukey’s honestly significant difference (HSD) test |
| Figure 4A | NbRab-G3c+GFP to GFP | 0.0089 | ** | Tukey’s honestly significant difference (HSD) test |
| Figure 4A | NbRab-G3c+Pi17063(0.05μM) to NbRab-G3c+Pi17063(0.1μM) | 0.4507 | ns | Tukey’s honestly significant difference (HSD) test |
| Figure 4A | NbRab-G3c+Pi17063(0.05μM) to NbRab-G3c+Pi17063(0.2μM) | 0.0663 | ns | Tukey’s honestly significant difference (HSD) test |
| Figure 4A | NbRab-G3c+Pi17063(0.05μM) to Pi17063 | 0.0077 | ** | Tukey’s honestly significant difference (HSD) test |
| Figure 4A | NbRab-G3c+Pi17063(0.05μM) to GFP | 0.004 | ** | Tukey’s honestly significant difference (HSD) test |
| Figure 4A | NbRab-G3c+Pi17063(0.1μM) to NbRab-G3c+Pi17063(0.2μM) | 0.1805 | ns | Tukey’s honestly significant difference (HSD) test |
| Figure 4A | NbRab-G3c+Pi17063(0.1μM) to Pi17063 | 0.0037 | ** | Tukey’s honestly significant difference (HSD) test |
| Figure 4A | NbRab-G3c+Pi17063(0.1μM) to GFP | 0.002 | ** | Tukey’s honestly significant difference (HSD) test |
| Figure 4A | NbRab-G3c+Pi17063(0.2μM) to Pi17063 | 0.001 | ** | Tukey’s honestly significant difference (HSD) test |
| Figure 4A | NbRab-G3c+Pi17063(0.2μM) to GFP | 0.0006 | *** | Tukey’s honestly significant difference (HSD) test |
| Figure 4A | Pi17063 to GFP | 0.4962 | ns | Tukey’s honestly significant difference (HSD) test |
| Figure 4F | NbRab-G3c+GFP to NbRab-G3c+Pi17063 | 0.0017 | ** | Tukey’s honestly significant difference (HSD) test |
| Figure 4F | NbRab-G3c+GFP to NbRab-G3c+Pi17063 M1 | 0.9712 | ns | Tukey’s honestly significant difference (HSD) test |
| Figure 4F | NbRab-G3c+GFP to NbRab-G3c+Pi17063 M2 | 0.0399 | * | Tukey’s honestly significant difference (HSD) test |
| Figure 4F | NbRab-G3c+GFP to NbRab-G3c+Pi17063 M3 | 0.2115 | ns | Tukey’s honestly significant difference (HSD) test |
| Figure 4F | NbRab-G3c+GFP to NbRab-G3c+Pi17063 M4 | 0.5998 | ns | Tukey’s honestly significant difference (HSD) test |
| Figure 4F | NbRab-G3c+GFP to Pi17063 | 0.0002 | *** | Tukey’s honestly significant difference (HSD) test |
| Figure 4F | NbRab-G3c+GFP to GFP | 0.0002 | *** | Tukey’s honestly significant difference (HSD) test |
| Figure 4F | NbRab-G3c+Pi17063 to NbRab-G3c+Pi17063 M1 | 0.0017 | ** | Tukey’s honestly significant difference (HSD) test |
| Figure 4F | NbRab-G3c+Pi17063 to NbRab-G3c+Pi17063 M2 | 0.0428 | * | Tukey’s honestly significant difference (HSD) test |
| Figure 4F | NbRab-G3c+Pi17063 to NbRab-G3c+Pi17063 M3 | 0.0086 | ** | Tukey’s honestly significant difference (HSD) test |
| Figure 4F | NbRab-G3c+Pi17063 to NbRab-G3c+Pi17063 M4 | 0.003 | ** | Tukey’s honestly significant difference (HSD) test |
| Figure 4F | NbRab-G3c+Pi17063 to Pi17063 | <0.0001 | *** | Tukey’s honestly significant difference (HSD) test |
| Figure 4F | NbRab-G3c+Pi17063 to GFP | <0.0001 | *** | Tukey’s honestly significant difference (HSD) test |
| Figure 4F | NbRab-G3c+Pi17063 M1 to NbRab-G3c+Pi17063 M2 | 0.0398 | * | Tukey’s honestly significant difference (HSD) test |
| Figure 4F | NbRab-G3c+Pi17063 M1 to NbRab-G3c+Pi17063 M3 | 0.2088 | ns | Tukey’s honestly significant difference (HSD) test |
| Figure 4F | NbRab-G3c+Pi17063 M1 to NbRab-G3c+Pi17063 M4 | 0.5908 | ns | Tukey’s honestly significant difference (HSD) test |
| Figure 4F | NbRab-G3c+Pi17063 M1 to Pi17063 | 0.0002 | *** | Tukey’s honestly significant difference (HSD) test |
| Figure 4F | NbRab-G3c+Pi17063 M1 to GFP | 0.0001 | *** | Tukey’s honestly significant difference (HSD) test |
| Figure 4F | NbRab-G3c+Pi17063 M2 to NbRab-G3c+Pi17063 M3 | 0.2656 | ns | Tukey’s honestly significant difference (HSD) test |
| Figure 4F | NbRab-G3c+Pi17063 M2 to NbRab-G3c+Pi17063 M4 | 0.0827 | ns | Tukey’s honestly significant difference (HSD) test |
| Figure 4F | NbRab-G3c+Pi17063 M2 to Pi17063 | <0.0001 | *** | Tukey’s honestly significant difference (HSD) test |
| Figure 4F | NbRab-G3c+Pi17063 M2 to GFP | <0.0001 | *** | Tukey’s honestly significant difference (HSD) test |
| Figure 4F | NbRab-G3c+Pi17063 M3 to NbRab-G3c+Pi17063 M4 | 0.41 | ns | Tukey’s honestly significant difference (HSD) test |
| Figure 4F | NbRab-G3c+Pi17063 M3 to Pi17063 | 0.0001 | *** | Tukey’s honestly significant difference (HSD) test |
| Figure 4F | NbRab-G3c+Pi17063 M3 to GFP | 0.0001 | *** | Tukey’s honestly significant difference (HSD) test |
| Figure 4F | NbRab-G3c+Pi17063 M4 to Pi17063 | 0.0001 | *** | Tukey’s honestly significant difference (HSD) test |
| Figure 4F | NbRab-G3c+Pi17063 M4 to GFP | 0.0001 | *** | Tukey’s honestly significant difference (HSD) test |
| Figure 4F | Pi17063 to GFP | 0.9298 | ns | Tukey’s honestly significant difference (HSD) test |
| Figure 6C | NbRab-G3c+GFP to NbRab-G3c+Pi17063 | 0.3306 | ns | Tukey’s honestly significant difference (HSD) test |
| Figure 6C | NbRab-G3c+GFP to NbRab-G3c+NbGYP | 0.0226 | * | Tukey’s honestly significant difference (HSD) test |
| Figure 6C | NbRab-G3c+GFP to Pi17063 | 0.0078 | ** | Tukey’s honestly significant difference (HSD) test |
| Figure 6C | NbRab-G3c+GFP to GFP | 0.0087 | ** | Tukey’s honestly significant difference (HSD) test |
| Figure 6C | NbRab-G3c+GFP to NbGYP | 0.0057 | ** | Tukey’s honestly significant difference (HSD) test |
| Figure 6C | NbRab-G3c+Pi17063 to NbRab-G3c+NbGYP | 0.0805 | ns | Tukey’s honestly significant difference (HSD) test |
| Figure 6C | NbRab-G3c+Pi17063 to Pi17063 | 0.0029 | ** | Tukey’s honestly significant difference (HSD) test |
| Figure 6C | NbRab-G3c+Pi17063 to GFP | 0.0033 | ** | Tukey’s honestly significant difference (HSD) test |
| Figure 6C | NbRab-G3c+Pi17063 to NbGYP | 0.0022 | ** | Tukey’s honestly significant difference (HSD) test |
| Figure 6C | NbRab-G3c+NbGYP to Pi17063 | 0.0005 | *** | Tukey’s honestly significant difference (HSD) test |
| Figure 6C | NbRab-G3c+NbGYP to GFP | 0.0006 | *** | Tukey’s honestly significant difference (HSD) test |
| Figure 6C | NbRab-G3c+NbGYP to NbGYP | 0.0004 | *** | Tukey’s honestly significant difference (HSD) test |
| Figure 6C | Pi17063 to GFP | 0.8195 | ns | Tukey’s honestly significant difference (HSD) test |
| Figure 6C | Pi17063 to NbGYP | 0.724 | ns | Tukey’s honestly significant difference (HSD) test |
| Figure 6C | GFP to NbGYP | 0.5768 | ns | Tukey’s honestly significant difference (HSD) test |

**Statistical analysis table of** **Rab-G expression pattern**

| **Figure** | **Sample** | ***P* Value** | **Significance** | **Test** |
| --- | --- | --- | --- | --- |
| Figure S9A | 0h to 3h | 0.8677 | ns | Tukey’s honestly significant difference (HSD) test |
| Figure S9A | 0h to 6h | 0.107 | ns | Tukey’s honestly significant difference (HSD) test |
| Figure S9A | 0h to 12h | 0.0193 | * | Tukey’s honestly significant difference (HSD) test |
| Figure S9A | 0h to 24h | 0.1587 | ns | Tukey’s honestly significant difference (HSD) test |
| Figure S9A | 0h to 48h | 0.0019 | ** | Tukey’s honestly significant difference (HSD) test |
| Figure S9A | 3h to 6h | 0.08 | ns | Tukey’s honestly significant difference (HSD) test |
| Figure S9A | 3h to 12h | 0.0141 | * | Tukey’s honestly significant difference (HSD) test |
| Figure S9A | 3h to 24h | 0.1201 | ns | Tukey’s honestly significant difference (HSD) test |
| Figure S9A | 3h to 48h | 0.0014 | ** | Tukey’s honestly significant difference (HSD) test |
| Figure S9A | 6h to 12h | 0.3565 | ns | Tukey’s honestly significant difference (HSD) test |
| Figure S9A | 6h to 24h | 0.8151 | ns | Tukey’s honestly significant difference (HSD) test |
| Figure S9A | 6h to 48h | 0.0468 | * | Tukey’s honestly significant difference (HSD) test |
| Figure S9A | 12h to 24h | 0.254 | ns | Tukey’s honestly significant difference (HSD) test |
| Figure S9A | 12h to 48h | 0.2326 | ns | Tukey’s honestly significant difference (HSD) test |
| Figure S9A | 24h to 48h | 0.0303 | * | Tukey’s honestly significant difference (HSD) test |

**Statistical analysis table of** **protein expression**

| **Figure** | **Sample** | **Difference between means** | **95% confidence interval** | ***P* Value** | **Significance** | **Test** |
| --- | --- | --- | --- | --- | --- | --- |
| Figure 4C | GFP to GFP-Pi17063 | 3.731 ± 0.9605 | 1.065 to 6.398 | 0.0178 | * | t test/two-tailed |
